# Tumor vasculature-targeted ^10^B delivery by an Annexin A1-binding peptide boosts effects of boron neutron capture therapy

**DOI:** 10.1186/s12885-020-07760-x

**Published:** 2021-01-15

**Authors:** Tohru Yoneyama, Shingo Hatakeyama, Mihoko Sutoh-Yoneyama, Taku Yoshiya, Tsuyoshi Uemura, Takehiro Ishizu, Minoru Suzuki, Shingo Hachinohe, Shintaro Ishiyama, Motohiro Nonaka, Michiko N. Fukuda, Chikara Ohyama

**Affiliations:** 1 Department of Glycotechnology, Center for Advanced Medical Research, Hirosaki University Graduate School of Medicine, 5-Zaifu-cho, Hirosaki, 036-8562 Japan; 2 Department of Urology, Hirosaki University Graduate School of Medicine, 5-Zaifu-cho, Hirosaki, 036-8562 Japan; 3 Department of Cancer Immunology and Cell Biology, Oyokyo Kidney Research Institute, 90 Kozawa Yamazaki, Hirosaki, 036-8243 Japan; 4 Peptide Institute Inc., 7-2-9 Saito-Asagi, Osaka, Ibaraki 567-0085 Japan; 5 Particle Radiation Oncology Research Center, Institute for Integrated Radiation and Nuclear Science (KURNS), Kyoto University, 2-1010 Asashiro-nishi, Kumatori-cho, Sennan-gun, Osaka, 590-0494 Japan; 6 Aomori Prefecture Quantum Science Center (QSC), 2-190 Omotedate, Obuchi, Rokkasho-mura, Kamikita-gun 039-3212 Japan; 7 Faculty of Science and Technology, Hirosaki University Graduate School of Science and Technology, 1-Bunkyo-cho, Hirosaki, 036-8562 Japan; 8 Department of Biological Chemistry, Human Health Sciences, Graduate School of Medicine, Kyoto University, 53 Shogoin-Kawahara-cho, Sakyo-ku, Kyoto, 606-8507 Japan; 9 Tumor Microenvironment and Cancer Immunology Program, NCI-Designated Cancer Center, Sanford Burnham Prebys Medical Discovery Institute, 10901 North Torrey Pines Road, La Jolla, CA 92037 USA

**Keywords:** Drug delivery, Peptide, Annexin A1, Tumor vasculature, Boron neutron capture therapy

## Abstract

**Background:**

*p*-Boronophenylalanine (^10^BPA) is a powerful ^10^B drug used in current clinical trials of BNCT. For BNCT to be successful, a high (500 mg/kg) dose of ^10^BPA must be administered over a few hours. Here, we report BNCT efficacy after rapid, ultralow-dose administration of either tumor vasculature-specific annexin A1-targeting IFLLWQR (IF7)-conjugated ^10^BPA or borocaptate sodium (^10^BSH).

**Methods:**

(1) IF7 conjugates of either ^10^B drugs intravenously injected into MBT2 bladder tumor-bearing mice and biodistribution of ^10^B in tumors and normal organs analyzed by prompt gamma-ray analysis. (2) Therapeutic effect of IF7-^10^B drug-mediated BNCT was assessed by either MBT2 bladder tumor bearing C3H/He mice and YTS-1 tumor bearing nude mice.

**Results:**

Intravenous injection of IF7C conjugates of either ^10^B drugs into MBT2 bladder tumor-bearing mice promoted rapid ^10^B accumulation in tumor and suppressed tumor growth. Moreover, multiple treatments at ultralow (10–20 mg/kg) doses of IF7-^10^B drug-mediated BNCT significantly suppressed tumor growth in a mouse model of human YTS-1 bladder cancer, with increased Anxa1 expression in tumors and infiltration by CD8-positive lymphocytes.

**Conclusions:**

We conclude that IF7 serves as an efficient ^10^B delivery vehicle by targeting tumor tissues via the tumor vasculature and could serve as a relevant vehicle for BNCT drugs.

**Supplementary Information:**

The online version contains supplementary material available at 10.1186/s12885-020-07760-x.

## Background

Boron neutron capture therapy (BNCT) is based on a nuclear fission reaction between nonradioactive isotope ^10^B atoms and low-energy thermal neutrons, which generates high linear energy transfer α particles and a recoiled lithium nucleus (^7^Li) that selectively destroy the DNA helix in tumor cells [1, 2]. For successful therapy, ^10^B must reside inside the targeted cancer cells, given that the α particles and Li nucleus generate high energy within a 10 μm radius, which is equivalent to the size of a single cell. Two boron-10 delivery agents, *p*-boronophenylalanine (^10^BPA) and borocaptate sodium (^10^BSH), have been used in clinical studies [3–5]. ^10^BPA is a phenylalanine analog actively transported into tumor cells mainly by an L-type amino acid transporter 1 (LAT1) overexpressed on the membrane of many cancer cells [6]. In these procedures ^10^BPA content in cancer cells is detected using positron emission tomography (PET) imaging with ^18^F-BPA [7]. However, although ^10^BPA accumulates in normal cells, it is not effective in populations of tumor cells that proliferate slowly. The other reagent, ^10^BSH, harbors twelve ^10^B atoms, making it an extremely efficient ^10^B carrier. ^10^BSH is used primarily to treat malignant gliomas as ^10^BSH passively accumulates only in regions containing tumors where the blood–brain barrier has been destroyed. Although ^10^BSH accumulates and is retained more efficiently in tumor regions compared to normal tissue [8], it is present only in intercellular spaces and not internalized by cells. Therefore, α particles and ^7^Li generated from ^10^BSH sometimes do not reach tumor cell DNA, minimizing the therapeutic effect of ^10^BSH-mediated BNCT.

To address limitations of ^10^BPA and ^10^BSH, several ^10^B delivery systems using therapeutic doses of ^10^BPA- or ^10^BSH-containing drugs have been developed. In the case of ^10^BPA, Nomoto et al. reported that poly(vinyl alcohol) (PVA)-^10^BPA reversible boronate esters in aqueous solution (PVA-^10^BPA) are internalized by cancer cells through LAT1-mediated endocytosis and then localize to endo−/lysosomes, enhancing cellular uptake and slowing untoward efflux. In a previous in vivo study comparing it with clinically-used fructose-^10^BPA complexes, PVA-^10^BPA exhibited efficient accumulation and prolonged retention in tumors with quick clearance from the bloodstream and normal organs [9]. By contrast, Iguchi et al. reported that ^10^BSH fused with a short arginine peptide (3R, ^10^BSH-3R) is internalized by cancer cells in vitro and in vivo [8]. Although these novel ^10^BPA and ^10^BSH pharmacophores have been used clinically as a second-generation boron compounds for BNCT, they must be administered at extremely high doses, and it takes several hours for the compound to reach a therapeutically effective ^10^B concentration in tumor cells.

Tumor angiogenesis is defined as formation of new blood vessels to support tumor growth and metastasis. Model mice null for annexin A1 (Anxa1) show significantly suppressed tumor growth due to lack of angiogenesis, suggesting that Anxa1 is essential for tumor vascularization [10]. Anxa1 is reportedly present on the surface of tumor endothelial cells in several tumor types in mice and humans [11–14] and has been proposed to be a valid target for the tumor vasculature [15–18]. Previously, we discovered the carbohydrate mimetic peptide IFLLWQR (IF7) using peptide-displaying phage technology. We administered IF7 conjugated to fluorescent Alexa 488 to tumor-bearing mice and demonstrated excellent targeting to Anxa1 within minutes of injection [15, 19]. We also showed that IF7 conjugated to the potent anticancer drug SN-38 (IF7C(RR)-SN38) and injected intravenously into nude mice carrying human colon HCT116 tumors efficiently suppressed tumor growth at 5% the dose level of SN-38 with no apparent side effects. Recently, we showed that IF7C(RR)-SN38 crosses the blood-brain-barrier and suppresses growth of brain tumors in mouse model and Solutol HS15-formulated IF7C(RR)-SN38 may have promoted an anti-tumor immune response [20]. We conclude that the specific Anxa1-binding IF7 peptide serves as highly efficient vehicle to deliver anticancer drugs to tumors in vivo.

Annexin family proteins localize to endothelial caveolae surfaces and are internalized through endocytosis [21]. Antibodies bound to endothelial caveoli proteins are reportedly efficiently transported to the basal surface and released to the stroma below [22]. Moreover, IF7C(RR)-conjugated poly-L-lysine undergoes similar apical-to-basal transport through endothelial cells in vitro and in vivo. Others have reported that when ^18^F-labeled IF7 peptides (^18^F-AIF-NOTA-IF7 or ^18^F-AI-NODA-Bn-p-SCN-GGGRDN-IF7) are intravenously injected into A431 epidermoid carcinoma-bearing mice, ^18^F-labeled IF7 rapidly accumulates in tumors (within 30 min) based on micro-PET imaging [16, 17]. Moreover, when DiR-labeled IF7-nanoparticles were intravenously injected into MCF-7/ADR tumor-bearing mice, in 1 h those nanoparticles had accumulated dramatically more rapidly in tissue than had DiR-labeled nanoparticles lacking IF7, based on in vivo imaging [18]. Therefore, ^18^F-labeled IF7 or DiR-labeled IF7-nanoparticles may also serve as a tracer candidate for tumor imaging. Here, we asked whether IF7 peptide can be utilized in current BNCT applications to reduce required doses and rapidly target ^10^BPA and ^10^BSH to tumors.

**Fig. 1:**
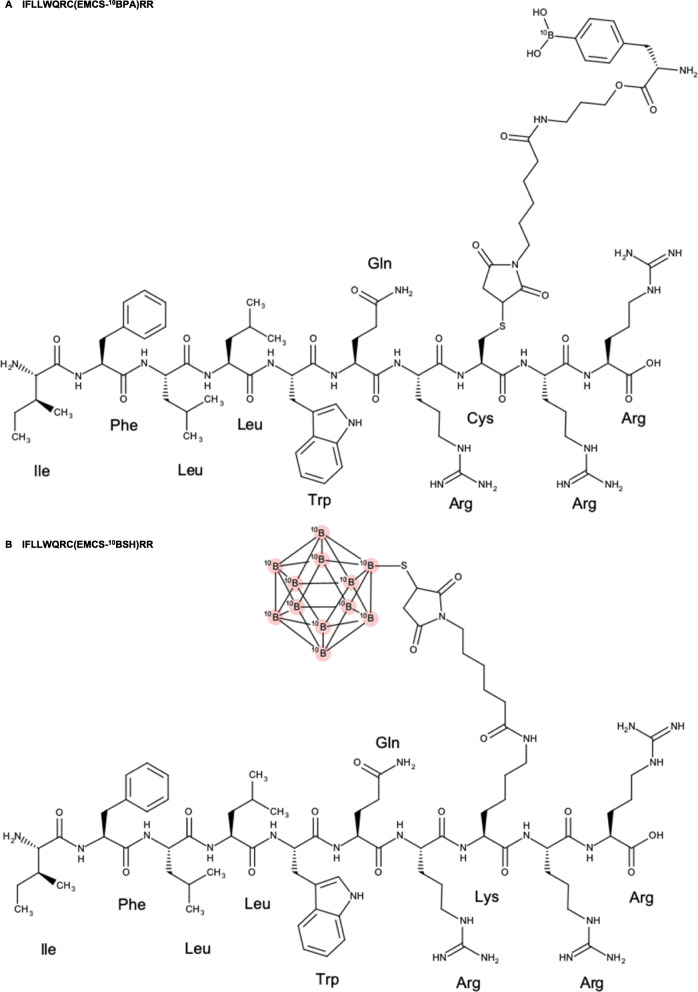
Structure of IF7C(^10^BPA)RR and IF7K(^10^BSH)RR. Shown are chemical structures of (**a**) IFLLWQRC(EMCS-^10^BPA)RR and (**b**) IFLLWQRK(EMCS-^10^BSH)RR

To test this hypothesis, we synthesized IF7-conjugated ^10^BPA or ^10^BSH (Fig. 1) for use in BNCT studies in vivo. We report that administration of an ultralow dose (10–20 mg/kg) of IF7C(^10^BPA)RR or IF7K(^10^BSH)RR to bladder tumor-bearing mice enhanced the ability of BNCT to induce rapid ^10^B accumulation in tumor tissues and significantly suppressed tumor growth with no apparent side effects.

## Methods

### General information

*p*-Boronophenylalanine (^10^BPA) and borocaptate sodium (^10^BSH) were purchased from Interpharma Praha A.s. (Praha, Czech Republic). Mouse anti-annexin A1 antibody (MC-16) was prepared by Dr. Motohiro Nonaka at Kyoto University. Anti-human Ki-67 antigen (clone MIB-1) antibody was purchased from Agilent Technologies Japan, Ltd. (Tokyo, Japan). Anti-mouse CD8α (EPR21769, ab217344) antibody and anti-mouse CD31 (EPR17259, ab182981) were purchased from Abcam PLC (Cambridge, UK). The hematoxylin histological staining reagent was purchased from Dako North America Inc. (Carpinteria CA, USA). Eosin alcohol solution, acid extract, and 20% formalin solution were purchased from Fujifilm Wako Pure Chemical Corporation Ltd. (Osaka, Japan). Teflon tubes with caps (14 φ × 44 L) were purchased from MonKiko Ltd. (Osaka, Japan). *N*-(6-maleimidocaproyloxy) sulfosuccinimide **(**Sulfo-EMCS) was purchased from Dojindo Laboratories (Kumamoto, Japan). Reagents and solvents not described above were obtained from Peptide Institute, Inc. (Osaka, Japan); FUJIFILM Wako Pure Chemical Corporation (Osaka, Japan); Tokyo Chemical Industry Co., Ltd. (Tokyo, Japan); Nacalai Tesque, Inc. (Kyoto, Japan); Watanabe Chemical Industries, Ltd. (Hiroshima, Japan); Merck KGaA (Darmstadt, Germany); and Sigma-Aldrich Co. LLC. (St. Louis, MO). Preparative HPLC was carried out on a Shimadzu liquid chromatograph model LC-8A (Kyoto, Japan) with a YMC-Pack ODS-A (30 × 250 mm), with the following solvents: 0.1% TFA in H_2_O and 0.1% TFA in CH_3_CN. Flow rate was 20 mL minute^− 1^ and detection at 220 nm. Analytical HPLC was performed on a Shimadzu liquid chromatograph prominence (Kyoto, Japan) with a Zorbax 300SB-C18 (4.6 × 150 mm) column using the following solvents: 0.1% TFA in H_2_O and 0.1% TFA in CH_3_CN. Flow rate was 1 mL per minute^− 1^ (40 °C) with detection at 220 nm. Mass spectra (MS) were observed with an Agilent G1946A LC/MSD detector using an Agilent 1100 series HPLC system; observed masses were calculated with experimental *m/z* values (most abundant masses) for each protonation state of the target peptide.

### Solid phase peptide synthesis (SPPS)

Automated peptide synthesis by Boc SPPS was performed on an ABI 430A peptide synthesizer (Applied Biosystems, CA, USA). The peptide chain was elongated on Pam-resin using the coupling protocol of Boc-amino acid/DCC/HOBt. The following side chain-protected amino acids were employed: Trp(For), Arg(Tos), Cys(MeBzl), and Lys(Fmoc). For a Lys (EMCS)-containing peptide, after construction of the protected peptide chain on the resin, Lys (Fmoc) was deprotected with 20% piperidine in NMP and reacted with EMCS to yield Lys (EMCS)-containing protected peptide resin. Peptides were cleaved/deprotected in HF–4-methylphenol (8:2) and purified by RP-HPLC before use in other experiments.

### IF7 peptide conjugation to ^10^B drugs

Conjugation of synthetic IF7 peptide to ^10^BPA (IF7C(^10^BPA)RR, Fig. 1a) or with ^10^BSH (IF7K(^10^BSH)RR, Fig. 1b) was performed by the Peptide Institute, Inc. (Osaka, Japan). Relevant to the former, EMCS-^10^BPA (189 mg) was attached to the peptide moiety (IFLLWQRCRR, 480 mg) prepared by SPPS in an acetate buffer, pH 8.0, (2 mL) for 1 h at 25 °C. Then, IF7C(^10^BPA)RR was purified by RP-HPLC and lyophilized to a white powder (584 mg), with the following characteristics: analytical HPLC: *t*_R_ = 11.3 min (15–65% CH_3_CN/0.1% TFA for 25 min) and purity: 99.5% (UV 220 nm detection). The molecular mass calculated for C_86_H_133_^10^BN_24_O_19_S is 1849.2, and the observed value was 1849.0 (Fig. S1A). In a different synthesis, ^10^BSH (121 mg) was attached to the peptide moiety (IFLLWQRK(EMCS)RR, 590 mg) in a manner similar to that reported above to yield IF7K(^10^BSH)RR as a white powder (411 mg), with the following characteristics: analytical HPLC: *t*_R_ = 11.4 min (20–70% CH_3_CN/0.1% TFA for 25 min) and purity: 98.8% (UV 220 nm detection). The molecular mass calculated for C_77_H_135_^10^B_12_N_23_O_15_S is 1775.3, and the observed value was 1775.1 (Fig. S1B).

### Cells, culture reagents, and animals

A murine MBT2 bladder cancer line was purchased from the Japanese Collection of Research BioResource cell bank (National Institute of Biomedical Innovation, Health and Nutrition, Tokyo, Japan). A human muscle invasive and high-grade bladder cancer cell line, YTS-1, was previously provided by Dr. Hiroshi Kakizaki (Yamagata University, Yamagata, Japan) [23, 24]. YTS-1 and MBT2 cells were maintained in RPMI-1640 medium (Fujifilm Wako Pure Chemical Corporation) supplemented with 10% fetal bovine serum (Thermo Fisher Scientific, Gibco, CA, USA) and 1% penicillin/streptomycin (Fujifilm Wako Pure Chemical Corporation) with 5% CO_2_ at 37 °C. Animals were obtained from CLEA Japan, Inc. (Tokyo, Japan). All animal studies were carried out in accordance with recommendations in the Guide for the Care and Use of Laboratory Animals of the National Institutes of Health. All protocols were approved by the Hirosaki University Graduate School of Medicine Animal Care and Use Committee (permit numbers: M17022 and M19021, https://www.innovation.hirosaki-u.ac.jp/kokai/kunren), Kyoto University Animal Care and Use Committee (permit numbers: #34 and #36, https://www.kyoto-u.ac.jp/ja/research/rule/ethic/arcku), and Aomori Prefecture Quantum Science Center Animal Care and Use Committee (permit numbers: DK001 and DK009, https://www.aomori-qsc.jp/research/animal.php). All surgeries were performed under anesthesia with 2% isoflurane inhalation, and all efforts were made to minimize suffering. All mice were sacrificed by cervical dislocation under anesthesia with 2% isoflurane inhalation. Same sex mice were housed together in individually ventilated cages with four or five mice per cage. All mice were maintained on a regular diurnal lighting cycle (12:12 light-dark) with ad libitum access to food (Radiation-sterilized diets CE-2, CLEA Japan) and water. Clean chip (CLEA Japan) was used bedding. Mice were housed under broken barrier-specific pathogen-free conditions in the Mouse Core Facility of Hirosaki University or the Institute for Integrated Radiation and Nuclear Science, Kyoto University or Aomori Prefecture Quantum Science Center.

### Determination of ^10^B concentration in tumors and normal organs by prompt gamma-ray analysis

MBT2 cells (1 × 10^6^ cells per mouse) plus 50 μl Matrigel (Corning Inc., NY, USA) were injected subcutaneously using a 27-gauge needle into the right thighs of 8-week-old female C3H/He mice under anesthesia with 2% isoflurane inhalation. The day of injection was defined as day 0. At 4 weeks after MBT2 cells injection, when MBT2 tumors were palpable, mice were randomly divided into four groups of 16 mice each and injected intravenously with: 1) fructose-^10^BPA ((0.791 mg/kg), 2) IF7C(^10^BPA)RR (7 mg/kg), 3) ^10^BSH 0.868 mg/kg), or 4) IF7K(^10^BSH)RR (7 mg/kg). Within 5, 10, 20, and 40 min of injection, four mice at each time point were sacrificed by cervical dislocation under anesthesia with 2% isoflurane inhalation. From each mouse, the tumor, brain, lung, heart, liver, kidney, bladder, stomach, intestine, spleen, skin, muscles, and blood were collected in a Teflon tube for ^10^B measurement. ^10^B concentrations in tissues were measured by prompt gamma-ray spectrometry using a thermal neutron guide tube installed at the Institute for Integrated Radiation and Nuclear Science, Kyoto University (KURNS).

### Treatment of MBT2 bladder tumor-bearing mice with nuclear reactor-based neutron capture therapy

MBT2 cells were injected as described above into the right thighs of 8-week-old female C3H/He mice, and the day of injection defined as day 0. Four weeks later, when MBT2 tumors were palpable, mice were randomly divided into six groups of 3 mice each: 1) untreated control, 2) IF7C(^10^BPA)RR, 3) IF7K(^10^BSH)RR, 4) neutron-irradiated controls, 5) IF7C(^10^BPA)RR-mediated BNCT, and 6) IF7K(^10^BSH)RR-mediated BNCT. In Groups 4, 5, and 6, tumors in right thighs were subjected to neutron beam irradiation at the heavy water facility of KURNS Research Reactor for 60 min at a power of 1 MW. Each mouse was held within an acrylic holder during neutron irradiation, and a LiF plate (50 mm thick) was used to shield the body from thermal neutrons, while exposing the tumor. Neutron fluences were measured by radioactivation of gold foils (3 mm diameter; 0.05 mm thick) on surface of both sides of the tumors. Since thermal neutrons were rapidly attenuated in the tumor, the average thermal neutron fluences of both sides were adopted as the fluence irradiated to the tumors. In this study, tumors were irradiated by 1.9 × 10^12^ thermal neutrons/cm^2^. Thermoluminescent dosimeters for γ-ray dosimetry were attached to tumor surfaces. The average γ-ray dose was 0.27 Gy. For groups 5 and 6, IF7C(^10^BPA)RR or IF7K(^10^BSH)RR was administered intravenously 40 min before neutron irradiation at a dose of 10 mg/kg. Tumor size was measured using calipers, and tumor volume (*V*) was calculated as: *V* = *ab*^*2*^/2, where *a* and *b* are the major and minor axes, respectively. At 3 weeks after BNCT, all mice were sacrificed by cervical dislocation under anesthesia with 2% isoflurane inhalation, and tumors weighed.

### Treatment of human YTS-1 xenograft mice with cyclotron accelerator-based neutron capture therapy to human YTS-1 xenograft

YTS-1 cells (2 × 10^6^ cells per mouse) plus 50 μl Matrigel (Corning Inc.) were injected subcutaneously using a 27-gauge needle into the right thighs of 8-week-old female BALB/c nu/nu mice under anesthesia with 2% isoflurane inhalation. The day of injection was defined as day 0. One week later, when YTS-1 xenografts were palpable, mice were randomly divided into six groups of 8 mice each: 1) untreated control, 2) IF7C(^10^BPA)RR, 3) IF7K(^10^BSH)RR, 4) neutron-irradiated controls, 5) IF7C(^10^BPA)RR-mediated BNCT, and 6) IF7K(^10^BSH)RR-mediated BNCT. In Groups 4, 5, and 6, tumors in thighs were subjected to neutron beam irradiation at the cyclotron-typed accelerator (Sumitomo Heavy Industries Ltd.) in the Aomori Prefecture Quantum Science Center for 60 min at a power of 100 mA at 20 MeV. Radiation of mice was performed as described above. The average of the γ-ray dose was 0.23 Gy. Groups 5 and 6, IF7C(^10^BPA)RR or IF7K(^10^BSH)RR was administered intravenously 40 min before irradiation at a dose of 20 mg/kg. One week after the first BNCT, the second was administered using both the same method and irradiation method. The tumor size and volume was calculated as above. Four weeks after the first BNCT, all mice were sacrificed by cervical dislocation under anesthesia with 2% isoflurane inhalation, and tumors were weighed and prepared for immunohistochemical analysis.

### Immunohistochemistry of YTS-1 xenografts

BNCT-treated YTS-1 tumors were collected as described above, fixed in 20% formalin solution and embedded in paraffin. Tissue sections 4 μm thick were mounted on silane-coated glass slides and air-dried for 1 h. Deparaffinized tissue sections underwent heat-induced epitope retrieval using a Histofine antigen retrieval reagent (pH 6.0) (Nichirei Biosciences Inc. Tokyo, Japan) and were then incubated with anti-human Ki-67 antigen (clone MIB-1, 1:2000 dilution) or mouse anti-Anxa1 monoclonal antibody (clone MC16, 1:100 dilution) in phosphate-buffered saline (PBS) containing 5% bovine serum albumin (BSA) at 4 °C overnight. Other sections were treated with heat-induced epitope retrieval with Histofine antigen retrieval reagent (pH 9.0) (Nichirei Biosciences Inc.) and then incubated with rabbit anti-mouse CD8α (antibody (EPR21769, 1:2000 dilution) or anti-mouse CD31 (EPR17259, 1:2000 dilution) in PBS containing 5% BSA at 4 °C overnight. The Envision/HRP rabbit mouse kit was used for antibody detection (Agilent Technologies Japan., Tokyo, Japan). Nuclear counterstaining was performed by incubating sections with hematoxylin solution (Agilent Technologies Japan) for 2 min at room temperature. Eosin alcohol solution (Fujifilm Wako Pure Chemical Corporation) was used to perform HE staining, according to the manufacturer’s instruction. Images (10× objective) were captured using a Keyence BZ-9000 fluorescence microscope (Keyence, Tokyo, Japan) and BZ-II analyzer Ver 2.2 (Keyence). The white balance was adjusted for each specimen.

### Cell counting protocols for YTS-1 xenografts

Six complete and non-overlapping tumor regions of interest (ROI) were selected from each case and saved as .tif files. The number of diaminobenzidine (DAB)-stained nuclei was determined in the ROI using color deconvolution and the particle analysis plug-in of the Fiji platform (ImageJ distribution, http://fiji.sc/Fiji). The mean number of CD8-positive lymphocytes in all six fields was used for statistical analysis. The number of diaminobenzidine (DAB)-stained blood vessels was counted in a ROI using the color deconvolution plug-in of the Fiji platform (ImageJ distribution, http://fiji.sc/Fiji). The mean number of CD31-positive blood vessels in all six fields was used for statistical analysis.

### Determination of the Ki-67 proliferation index for YTS-1 xenografts

As above, six complete and non-overlapping tumor regions of interest (ROI) were selected and saved as .tif files, and diaminobenzidine (DAB)- and hematoxylin-stained nuclei were counted. Ki-67-positive nuclei were included in the count, regardless of staining intensity, in line with recommendations of the International Ki-67 in Breast Cancer Working Group [25]. The number of DAB-stained nuclei was divided by the sum of DAB- and hematoxylin-stained nuclei, and values were expressed as a percentage. The mean percentage of Ki-67-positive cells in all six field was used for statistical analysis.

### Statistical analysis

Body weight, tumor volume, and measurement of ^10^B concentrations were obtained in vivo. The Ki-67 proliferation index was assessed as mean ± SD. All statistical calculations were performed using Graphpad Prism 8 (GraphPad, San Diego, CA, USA). For a non-normally distributed model, the Mann–Whitney *U*-test was used to analyze intergroup differences, while the Kruskal–Wallis test was used to analyze multiple group differences. A two-way analysis of variance test was used to analyze ^10^B concentrations in tissue and tumor volume with post hoc analysis. *P* values less than 0.05 were considered significant.

## Results

### Synthesis of IF7C(^10^BPA)RR and IF7K(^10^BSH)RR

To facilitate esterase-aided ^10^BPA release following delivery to a tumor, we conjugated IF7C to ^10^BPA via an ester bond with a propanolamine linker. Following analysis of 584 mg of IF7C(^10^BPA)RR, we determined its purity to be 99.5% (Fig. S1A). By contrast, to design a conjugate to be internalized by tumor cells, we conjugated IF7K to ^10^BSH directly through an uncleavable linker, *N*-(6-maleimidocaproyloxy) sulfosuccinimide **(**Sulfo-EMCS). We synthesized a total of 411 mg IF7K(^10^BSH)RR, and determined its purity to be 98.8% (Fig. S1B). Note that since IF7 peptide is poorly soluble in aqueous solution, for both syntheses we added two arginines (RR) to respective IF7C or IF7K C-termini to increase solubility [15].

### Quantitative analysis of ^10^B concentration in murine MBT2 tumors after prompt gamma-ray irradiation

**Fig. 2:**
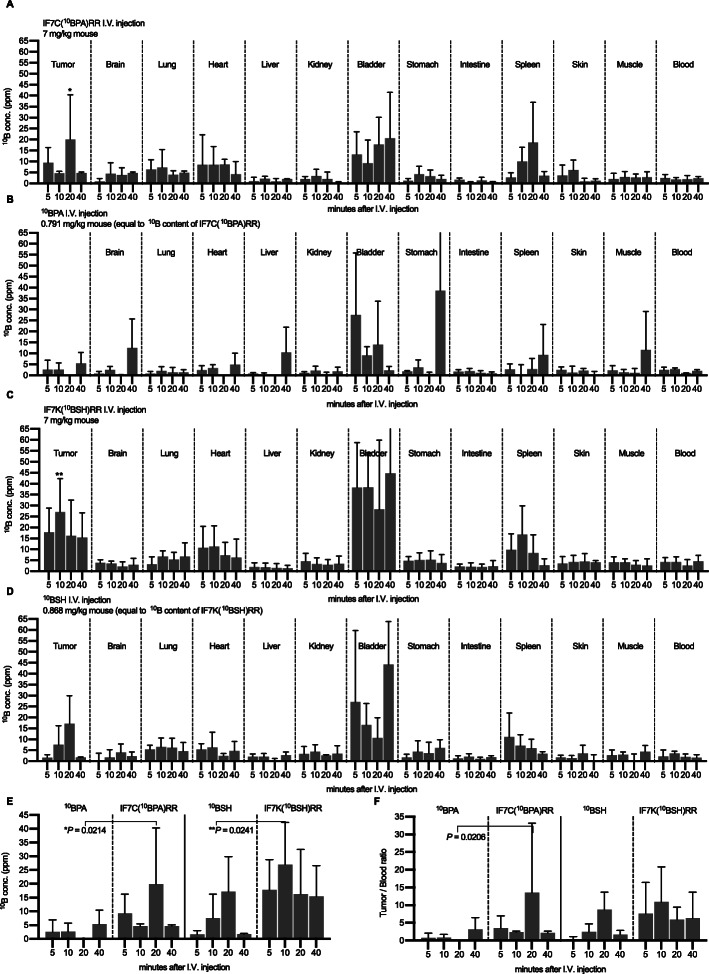
Determination of ^10^B concentration in tumors and normal organs by prompt gamma-ray analysis. ^10^B concentration in tumors and indicated normal organs from murine MBT2 tumor-bearing mice after ^10^B drug injection. Results are expressed as means ± SD (*n* = 3). ^10^B concentration following (**a**) IF7C(^10^BPA)RR administration, (**b**) ^10^BPA administration, (**c**) IF7K(^10^BSH)RR, (**d**) ^10^BSH administration, and (**e**) in tumors after injection of indicated reagents. (**f**) ^10^B tumor/blood ratio following administration of indicated reagents. Results are expressed as means ± SD. *P < 0.05 of ^10^BPA vs IF7C(^10^BPH)RR. **P < 0.05 of ^10^BSH vs IF7K(^10^BSH)RR (Holm–Sidak method)

To assess the timing of neutron irradiation for our in vivo BNCT study, we first compared intratumoral ^10^B accumulation after intravenous administration of both conventional ^10^B drugs versus IF7-^10^B drugs to mice. To do so, we intravenously administered Fructose-^10^BPA, ^10^BSH, IF7C(^10^BPA)RR, or IF7K(^10^BSH)RR into murine MBT2 bladder tumor-bearing C3H/He mice, and quantified ^10^B concentration in various organs after performing prompt gamma-ray analysis. As shown in Fig. 2a–f, intratumoral ^10^B concentration of IF7C(^10^BPA)RR- or IF7K(^10^BSH)RR-injected mice increased within 5 to 20 min. Intratumoral ^10^B concentrations of the IF7C(^10^BPA)RR group at 5 min (mean ± SD 9.3 ± 6.9 ppm), 10 min (4.6 ± 0.8 ppm), 20 min (19.9 ± 20.4 ppm), and 40 min (4.7 ± 0.5 ppm) after injection were higher than those in the^10^BPA group at 5 (2.5 ± 4.4 ppm), 10 (2.6 ± 3.1 ppm), 20 (0.0 ± 0.0 ppm), and 40 (5.4 ± 5.0 ppm) minutes after injection (Fig. 2a, b, and e). Intratumoral ^10^B concentrations of the IF7K(^10^BSH)RR group at 5 (17.8 ± 11.1 ppm), 10 (27.0 ± 15.3 ppm), 20 (16.2 ± 16.3 ppm), and 40 (15.4 ± 11.2 ppm) minutes after injection were higher than those of the ^10^BSH group at 5 (1.6 ± 1.4 ppm), 10 (7.5 ± 8.7 ppm), 20 (17.1 ± 12.8 ppm), and 40 (1.7 ± 0.3 ppm) minutes after injection (Fig. 2c, d and e).

Tumor/blood (T/B) ratios of IF7C(^10^BPA)RR and IF7K(^10^BSH)RR groups at 5 min (mean ± SD: 3.48 ± 3.49 and 7.61 ± 8.81, respectively), 10 min (2.41 ± 0.27 and 10.92 ± 9.89, respectively), 20 min (13.54 ± 19.68 and 5.89 ± 3.57, respectively), and 40 min (2.15 ± 0.50 and 6.28 ± 7.45, respectively) after injection were higher than those of the ^10^BPA or ^10^BSH groups at 5 (0.77 ± 1.33 and 0.41 ± 0.72, respectively), 10 (0.87 ± 0.88 and 2.46 ± 2.28, respectively), 20 (0.0 ± 0.0 and 8.72 ± 4.98, respectively), and 40 (3.16 ± 3.35 and 1.71 ± 1.14, respectively) minutes after injection (Fig. 2f). We concluded that the best time point to perform BNCT was within 40 min of injection.

### Effect of IF7-^10^B-mediated BNCT treatment on murine MBT2 tumor growth

**Fig. 3:**
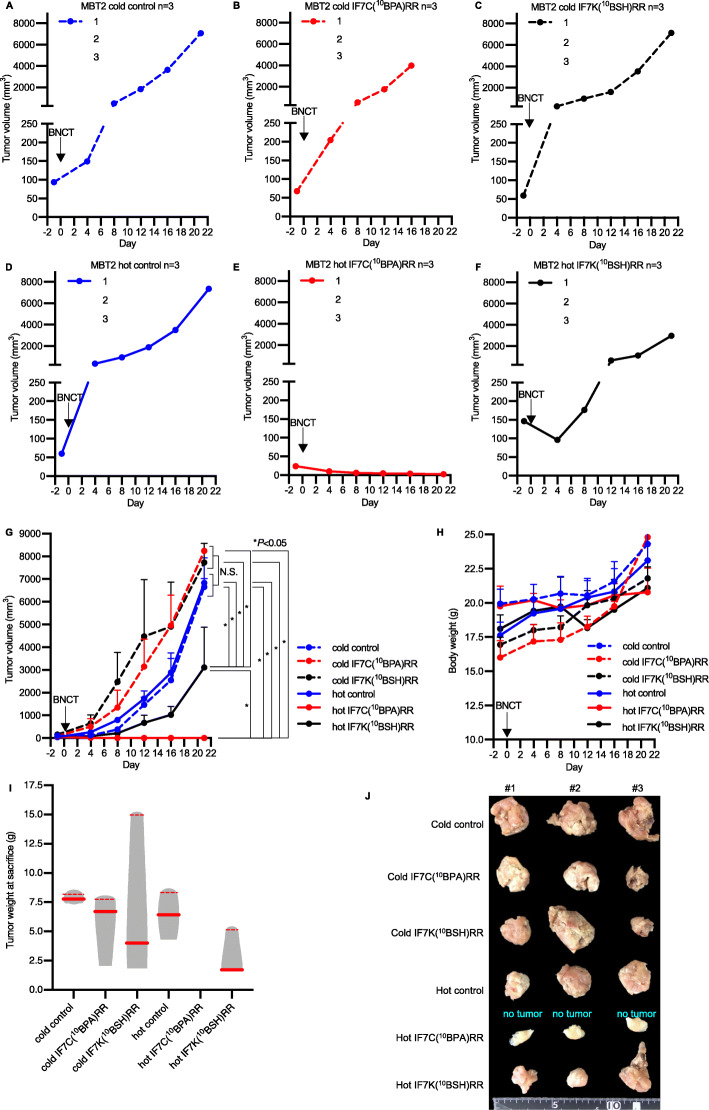
Antitumor effect of BNCT in murine MBT2 tumor-bearing mice. **a-f** Tumor growth curves from the following treatment groups: (**a**) untreated control (cold control, blue dashed line); (**b**) IF7C(^10^BPA)RR injection (cold IF7C(^10^BPA)RR, red dashed line); (**c**) IF7K(^10^BSH)RR injection (cold IF7K(^10^BSH)RR, black dashed line); (**d**) Neutron-irradiation (hot control, blue solid line); (**e**) IF7C(^10^BPA)RR-mediated BNCT (hot IF7C(^10^BPA)RR, red solid line); and **(f)** IF7K(^10^BSH)RR-mediated BNCT (hot IF7K(^10^BSH)RR, black solid line). Groups were intravenously injected, and tumors irradiated with epi/thermal neutrons 40 min after injection on day 1. **g** Tumor growth curve comparing groups analyzed in A-F. Results are expressed as means ± SD. *P < 0.05 (Holm–Sidak method). N.S.: no significant difference. **h** Body weight of indicated groups. Results are expressed as means ± SD. **i** Tumor weight of indicated groups at sacrifice. Results are expressed as violin plots with dot plots. Red bold lines indicate the median value, while red dashed lines indicate the interquartile range value (Mann–Whitney test) (**j**) Photograph of resected tumors from the injected right thighs. If a tumor completely shrank, whole right thighs were resected and labeled as “no tumor”

To evaluate a potential growth suppressive effect of IF7-^10^B drug-mediated BNCT, we performed an initial experiment (*n* = 3 each) using murine MBT2 bladder tumor-bearing C3H/He mice. MBT2 tumor size in six experimental groups of the model shown in Fig. 3a–f was monitored for up to 3 weeks after BNCT. Mice in 4 of the groups, namely, untreated control mice, IF7C(^10^BPA)RR-treated mice, IF7K(^10^BSH)RR-treated mice, and neutron-irradiated control mice (Fig. 3a–d), showed rapid tumor growth, and mean ± SD tumor volume in those groups by week 3 of the experiment was 6645 ± 372 mm^3^, 7728 ± 847 mm^3^, 8240 ± 0.0 mm^3^, and 6829 ± 1102 mm^3^, respectively. However, tumors subjected to IF7C(^10^BPA)RR or IF7K(^10^BSH)RR-mediated BNCT (Fig. 3e and f) showed markedly reduced tumor progression by 3 weeks after BNCT, and average tumor volume at that time point was 2.90 ± 0.49 mm^3^ or 3111 ± 1769 mm^3^, respectively. When we evaluated mice at days 16 and 21 after BNCT, differences in tumor volume between groups shown in Fig. 3e and f and those shown in Fig. 3a–d were significant (Fig. 3g, Table S1) (*P* < 0.05). However, we observed no significant differences in body weight between groups (Fig. 3h). Assessment of tumor weight and macroscopic observation of surgically removed tumors at sacrifice indicated smaller tumors in the IF7C(^10^BPA)RR-mediated BNCT groups (median [interquartile range: IQR] 0.000 g [0.000–0.000]) and IF7K(^10^BSH)RR-mediated BNCT groups (1.716 g [1.590–5.136]) relative to untreated control mice (7.779 g [7.419–8.185]), IF7C(^10^BPA)RR-treated (6.718 g [2.066–7.805]), IF7K(^10^BSH)RR-treated group (4.019 g [1.862–15.000]), and neutron-irradiated control group (6.431 g [4.324–8.370]) (Fig. 3i and j). However, these differences were not statistically significant possibly due to small sample size.

### Effect of IF7-^10^B-mediated BNCT treatment on growth of human YTS-1 xenografts

**Fig. 4:**
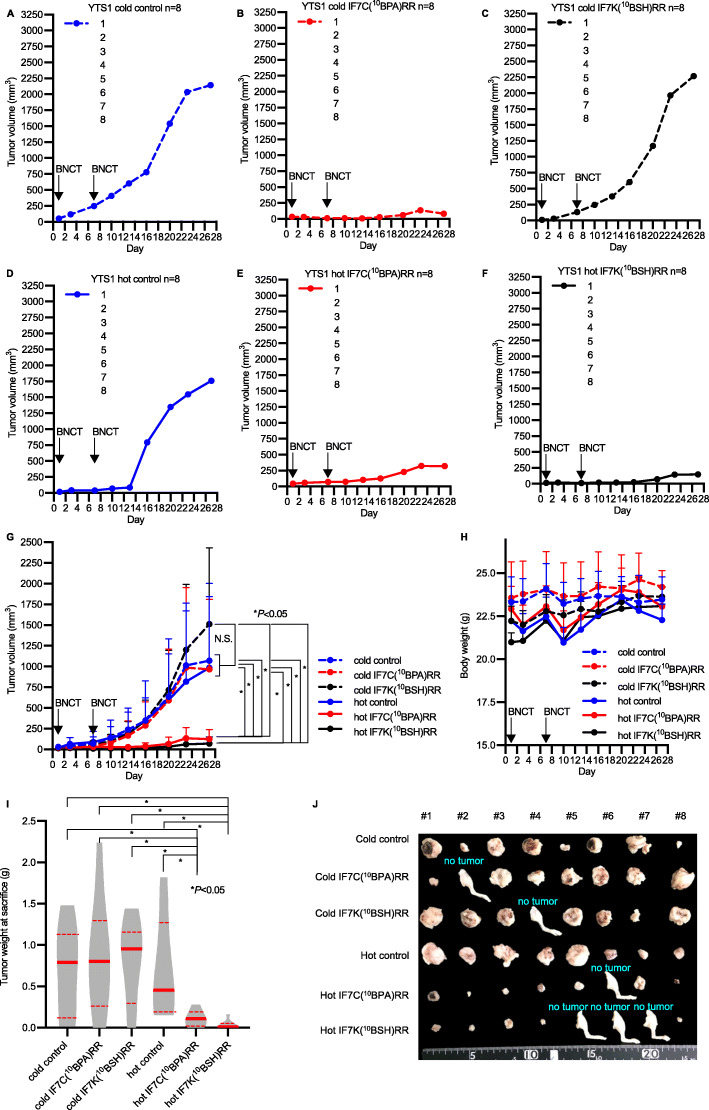
Antitumor effect of BNCT in a human YTS-1 xenograft model. **a-f** Tumor growth curves from the following treatment groups: (**a**) untreated control (cold control, blue dashed line); (**b**) IF7C(^10^BPA)RR injection (cold IF7C(^10^BPA)RR, red dashed line); (**c**) IF7K(^10^BSH)RR injection (cold IF7K(^10^BSH)RR, black dashed line); (**d**) Neutron-irradiation (hot control, blue solid line); (**e**) IF7C(^10^BPA)RR-mediated BNCT (hot IF7C(^10^BPA)RR, red solid line); and (**f**) IF7K(^10^BSH)RR-mediated BNCT (hot IF7K (^10^BSH)RR, black solid line). Indicated samples were intravenously injected, and tumors irradiated with epi/thermal neutrons 40 min after injection on days 1 and 7. **g** Tumor growth curve summarizing groups shown in A-F. Results are expressed as means ± SD. *P < 0.05 (Holm–Sidak method). N.S.: no significant difference. **h** Body weight of indicated groups. Results are expressed as means ± SD. **i** Tumor weight of indicated groups at sacrifice. Results are expressed as violin plots with dot plots. Red bold lines indicate the median value, while red dashed lines indicate the interquartile range. *P < 0.05 (Mann–Whitney test). (**j**) (**j**) Photograph of resected tumors from injected right thighs. If a tumor completely shrank, whole right thighs were resected and labeled as “no tumor”

We then assessed potential tumor growth suppression by IF7-^10^B drug-mediated BNCT in a larger cohort (*n* = 8 each) of nude mice bearing human YTS-1 bladder tumors by monitoring xenograft size in the six treatment groups named above for up to 4 weeks (Fig. 4). To do so, we performed two BNCT treatments administered with a one-week interval. Untreated control, IF7C(^10^BPA)RR-treated, and IF7K(^10^BSH)RR-treated mice showed rapid xenograft growth after the second BNCT, and mean ± SD tumor volumes at 4 weeks after the start of the first BNCT were 1069 ± 773 mm^3^, 965 ± 844 mm^3^, and 1511 ± 921 mm^3^, respectively. Mice subjected to neutron irradiation (Fig. 4a–d) showed slightly slower xenograft progression, with an average tumor volume of 983 ± 1020 mm^3^ by 4 weeks. However, when xenografts were subjected to IF7C(^10^BPA)RR- or IF7K(^10^BSH)RR-mediated BNCT (Fig. 4e and f), tumor progression was markedly reduced by 2 weeks after the second BNCT, and average tumor volumes at 4 weeks were 123 ± 114 mm^3^ or 69 ± 79 mm^3^, respectively. Differences in tumor volume between groups shown in Fig. 4e and f and Fig. 4a–d at days 20, 23, and 27 after BNCT treatment were significant (Fig. 4g, Table S2) (*P* < 0.05), although body weight was comparable between groups (Fig. 4h). Tumor weights of the IF7C(^10^BPA)RR- and IF7K(^10^BSH)RR-mediated BNCT groups (median [IQR]: 0.110 g [0.015–0.190] and 0.010 g [0.000–0.058], respectively) at sacrifice were significantly less than those seen in untreated control mice (0.790 g [0.113–1.130]), the IF7C(^10^BPA)RR-treated group (0.800 g [0.263–1.293]), the IF7K(^10^BSH)RR-treated group (0.955 g [0.295–1.155]), and the neutron-irradiated control group (0.455 g [0.195–1.273]) (Fig. 4i). Macroscopic observation of surgically removed tumors at sacrifice showed significantly smaller tumors in the IF7C(^10^BPA)RR- and IF7K(^10^BSH)RR-mediated BNCT groups compared with those in the other four groups (Fig. 4j).

### Immunohistochemical analysis of human YTS-1 xenografts

**Fig. 5:**
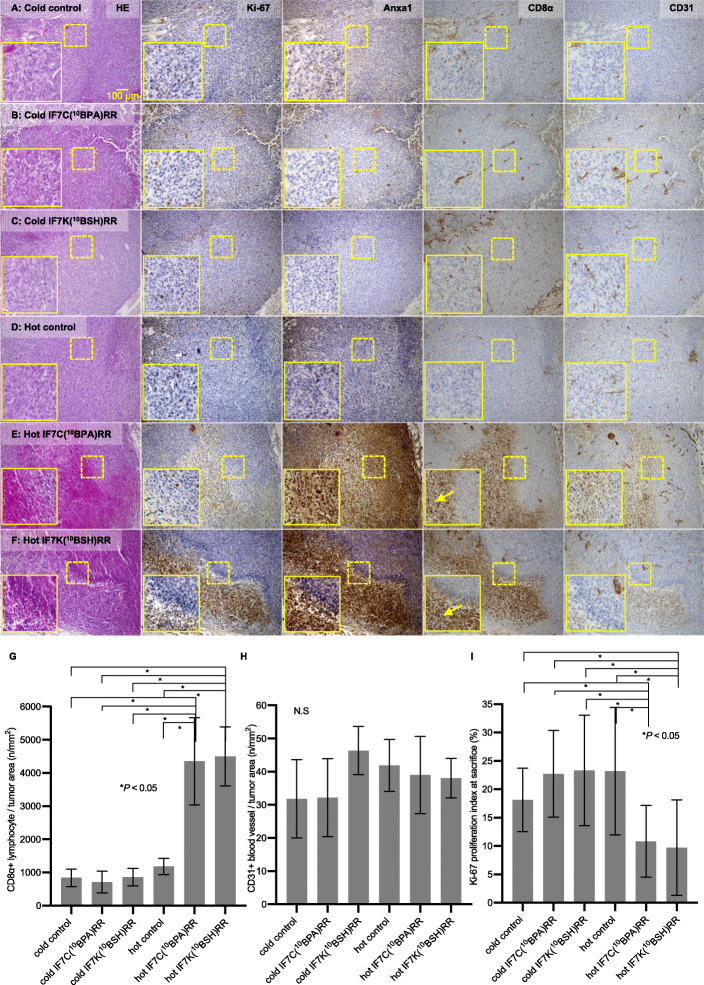
Immunohistochemical analysis of YTS-1 xenograft tumors. **a-f** Individual tumor tissue sections from the following groups: (**a**) no treatment (cold control); (**b**) IF7C(^10^BPA)RR injection (cold IF7C(^10^BPA)RR); (**c**) IF7K(^10^BSH)RR injection (cold IF7K(^10^BSH)RR); (**d**) Neutron irradiation (hot control); (**e**) IF7C(^10^BPA)RR-mediated BNCT (hot IF7C(^10^BPA)RR); and (**f**) IF7K(^10^BSH)RR-mediated BNCT (hot IF7K(^10^BSH)RR). Tissues were stained with HE (left column) or indicated antibodies. Yellow square indicated enlarged images. Yellow arrows indicate CD8α-positive lymphocytes. **g** The number of CD8+ lymphocytes infiltrating a given tumor area (n/mm^2^) in indicated groups. Results are expressed as means ± SD. *P < 0.05 (Kruskal–Wallis test). **h** The number of CD31+ blood vessels in a given tumor area (n/mm^2^) of indicated groups. Results are expressed as means ± SD. N.S.: no significant difference (Kruskal–Wallis test). **(i**) Ki-67 proliferation index of indicated groups. Results are expressed as means ± SD. *P < 0.05 (Kruskal–Wallis test)

At 3 weeks after the second BNCT treatment, we examined YTS-1 xenograft tissue samples from each group histologically (Fig. 5a–f) using standard hematoxylin and eosin (HE) staining after formalin fixation. Although histology of the untreated control mice, IF7C(^10^BPA)RR-treated mice, IF7K(^10^BSH)RR-treated mice, and neutron-irradiated control did not differ significantly, both the IF7C(^10^BPA)RR- and IF7K(^10^BSH)RR-mediated BNCT groups (Fig. 5e and f) showed tissue necrosis with infiltration of CD8α-positive lymphocytes (Fig. 5g), and the number of CD8-positive lymphocytes per tumor area of the IF7C(^10^BPA)RR- and IF7K(^10^BSH)RR-mediated BNCT groups (mean ± SD 4351 ± 1318 n/mm^2^, 4498 ± 890 n/mm^2^) (Fig. 5e and f) was significantly higher than numbers determined in untreated control mice, IF7C(^10^BPA)RR-treated mice, IF7K(^10^BSH)RR-treated mice (Fig. 5), a–cor irradiated control mice (Fig. 5d) (mean 840 ± 260 n/mm^2^, 709 ± 328 n/mm^2^, 860 ± 262 n/mm^2^, 1181 ± 244 n/mm^2^, respectively, *P* < 0.05). CD31-positive blood vessels were evident in tumor tissue of all groups (Fig. 5a–f), and the number of CD31-positive vessels per tumor area did not differ significantly among groups (Fig. 5h). The number of Ki-67-positive tumor cells following IF7C(^10^BPA)RR-mediated- or IF7K(^10^BSH)RR-mediated BNCT (Fig. 5e and f) significantly decreased relative to numbers seen in untreated control mice, IF7C(^10^BPA)RR-treated mice, IF7K(^10^BSH)RR-treated mice (Fig. 5a–c), or irradiated controls (Fig. 5d). The Ki-67 proliferation index following IF7C(^10^BPA)RR-mediated- or IF7K(^10^BSH)RR-mediated BNCT (mean ± SD 10.82 ± 6.31%, 9.73 ± 8.39%, respectively, *P* < 0.05) (Fig. 5i) also significantly decreased relative to that seen in untreated control mice, IF7C(^10^BPA)RR-treated mice, IF7K(^10^BSH)RR-treated mice, or irradiated controls (mean ± SD 18.11 ± 5.59%, 22.73 ± 7.64%, 23.32 ± 9.74%, respectively, *P* < 0.05). Anxa 1 expression in the IF7C(^10^BPA)RR-mediated or IF7K(^10^BSH)RR-mediated BNCT groups (Fig. 5e and f) was significantly higher than that seen in untreated control mice, IF7C(^10^BPA)RR-treated mice, IF7K(^10^BSH)RR-treated mice (Fig. 5a–c), or neutron-irradiated controls (Fig. 5d) at 3 weeks after the second BNCT. Overall, these IHC studies suggest that IF7-^10^B drug-mediated BNCT suppresses bladder tumor progression in mice by 3 weeks after the second treatment.

## Discussion

Clinically, boron-10 concentration in tumor tissues should exceed 25 ppm to achieve successful BNCT therapeutic outcomes. Although ^10^BPA is powerful ^10^B delivery drug that has been used in current clinical trials for BNCT, it must be administered at a extremely high dose (500 mg/kg) and requires a few hours to accumulate at tumor sites. Many researchers have tried to induce more effective intratumoral ^10^B accumulation using clinically effective doses of ^10^B drugs, but achieving this goal has been challenging. Here, we devised a novel delivery approach using the short 7-mer IF7 peptide, which is easily synthesized and can be readily modified. IF7 is, however, degraded by proteases in plasma and thus would not be antigenic, minimizing concerns regarding immune reactions. In our previous study using IF7-based chemotherapy, we were able to significantly reduce the dose of a conjugated anticancer drug, and the intravenously-injected IF7 construct reached tumor tissue within a minute [15]. These characteristics prompted us to evaluate the IF7 system as suitable for low dose and rapid delivery of ^10^B.

For those purposes, we delivered low doses of ^10^BPA and ^10^BSH by targeting respective IF7C(^10^BPA)RR and IF7K(^10^BSH)RR constructs to the tumor vasculature. In our biodistribution study of an ultralow dose (7 mg/kg) of these constructs to MBT2 tumor-bearing mice, intratumoral concentrations of IF7C(^10^BPA)RR or IF7K(^10^BSH)RR reached 20 or 25 ppm, respectively, concentrations higher those seen following administration of conventional ^10^BPA or ^10^BSH (Fig. 2). We observed that intratumoral ^10^B concentration in the IF7K(^10^BSH)RR-administered group reached 15 to 25 ppm between 5 and 40 min after injection of an ultralow dose. The tumor/blood (T/B) ratio of IF7K(^10^BSH)RR (T/B ratio: 5.89–10.92) and IF7C(^10^BPA)RR (T/B ratio: 2.15–13.54) by 40 min after injection was significantly higher than that of ^10^BSH (T/B ratio: 0.41–8.72) or ^10^BPA (T/B ratio: 0.00–3.16), suggesting that IF7 rapidly accumulates tumor tissues via tumor vasculature and that administration of an extremely high dose of conventional ^10^B drugs is not required. In vivo, the peptide moiety of the conjugate is likely digested by proteases, allowing ^10^B drug to freely penetrate tumor cells. This hypothesis is consistent with our previous histological observations showing that cells located around the vasculature undergo apoptosis and necrosis in tumor-bearing mice injected with IF7-geldanamycin [15].

Protease susceptibility is generally considered a disadvantage of peptide-based therapeutics [26, 27]. We also previously demonstrated that proteases in mouse plasma can alter the pharmacokinetics of IF7C(RR)-SN38 and IF7C-SN38 [15]. Given that IF7 has been demonstrated to deliver drugs to tumors, we conclude that the peptide moiety of IF7-conjugated ^10^B drugs remains intact until constructs reach the tumor vasculature, where they can then be degraded proteolytically. Our finding that IF7K(^10^BSH)RR and IF7C(^10^BPA)RR exhibit antitumor activities at considerably lower doses than those required in the absence of IF7 supports this assumption (Figs. 3 and 4).

The efficacy of IF7-conjugated ^10^B drugs also depends on the chemistry of conjugation. Here, we used an esterase-resistant linker for ^10^BSH and an esterase-cleavable linker for ^10^BPA, reasoning that ^10^BSH cannot be internalized by cells but BPA is internalized via the LAT1 transporter. Previously, we reported that when IF7C(RR)-SN38 with an esterase-cleavable linker was incubated at 37 °C with mouse plasma, 50% of SN38 was released from the conjugate within 10 min. As it takes 9 min for IF7 to target a tumor, these findings suggest that tumor growth suppression occurs when IF7C(RR)-SN38 remains intact in the initial 10-min window after intravenous injection [15]. When IF7-conjugated ^10^B drugs (Figs. 2, 3 and 4) are administered, it also takes 5 min for ^10^B to target the tumor tissue, suggesting that tumor growth is suppressed when IF7-^10^B drugs survive an initial 5-min window after injection. Although IF7-^10^BPA may be more stable in human plasma, which exhibits weaker esterase activity than mouse plasma, future studies should determine additional methods to enhance circulating drug stability and promote efficient drug release in tumor tissues.

**Fig. 6:**
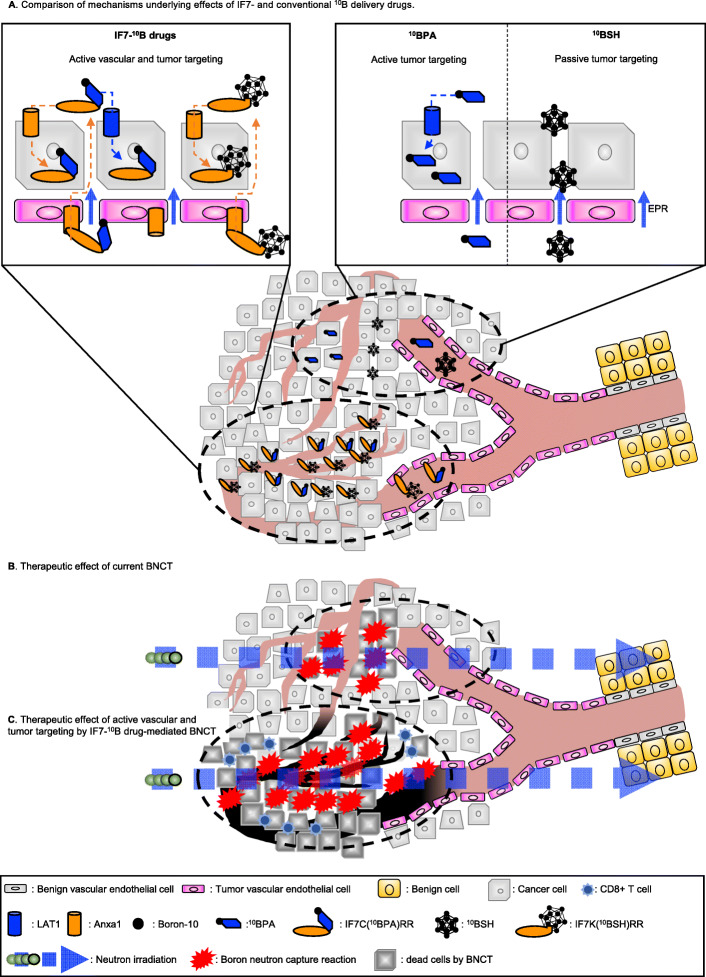
Schematic showing proposed boost in therapeutic effect by IF7-^10^B drug mediated BNCT. **a** IF7-^10^B drugs are actively transported into tumor vascular endothelial or tumor cells by annexin A1 (Anxa1) expressed on the membrane of both cells types. Successful BNCT requires a ultralow dose (20 mg/kg) of IF7-^10^B drugs administered within 20 min. ^10^BPA is actively transported into tumor cells mainly by an L-type amino acid transporter 1 (LAT1) overexpressed in the membrane of many cancer cells. Successful BNCT requires a very high dose (500 mg/kg) of ^10^BPA administered over a few hours. ^10^BSH harbors 12 ^10^B atoms and is an efficient ^10^B carrier. ^10^BSH accumulates efficiently and shows enhanced permeability and retention (EPR) in tumors relative to normal tissue and is present only in intercellular spaces and not internalized by cells. **b** Therapeutic effects of current BNCT. ^10^B-containing cancer cells are effectively killed by neutron irradiation, but no cytotoxic effect is seen in the tumor vasculature. **c** Proposed therapeutic effect of active vascular and tumor targeting by IF7-^10^B drug-mediated BNCT. We propose that BNCT treatment upregulates Anxa1 in tumor tissues due to an inflammatory response or induced immunogenicity, and that more effective ^10^B accumulation in tumor tissues occurs following a second injection of IF7-^10^B drugs. IF7-^10^B drug-mediated BNCT likely destroys the Anxa1-positive tumor vasculature, boosting its therapeutic potential. Thus, multiple ultralow doses of IF7-^10^B drug-mediated BNCT may be required to maximize the therapeutic effect

Previously, we demonstrated that IF7 binds to the Anxa1 N-terminus [15]. Annexins exhibit N-termini unique to each family member and an evolutionarily conserved core domain, and the Anxa1 N-terminal amino acid sequence is completely in mouse and humans [28], suggesting that IF7 would bind human Anxa1 expressed in the tumor vasculature. In this study, we performed an in vivo BNCT experiment using mice bearing either murine or human bladder tumors. In a previous preliminary small cohort study in murine MBT2 tumor-bearing mice, IF7-^10^B-mediated BNCT performed at a dose of 10 mg/kg significantly suppressed tumor growth (Fig. 3). Here, to exert stronger antitumor activity, we increased that dose to 20 mg/kg of IF7-^10^B drugs and performed two BNCT treatments in mice bearing human YTS-1 bladder tumors (Fig. 4). Our immunohistochemical study of human YTS-1 xenografts showed that the Ki-67 proliferation index significantly decreased in IF7-^10^B drug-mediated BNCT groups relative to that seen in non-irradiated groups by 3 weeks after the second BNCT treatment (Fig. 5). In addition, HE and CD8α staining of samples from IF7-^10^B drug-mediated BNCT groups indicated tissue necrosis accompanied by infiltration of CD8α-positive lymphocytes (Fig. 5g). It is well known that nude mice exhibit residual numbers of T cells as well as high numbers of NK and other immune cells [29, 30], and it is also reported that levels of CD8-positive T cells increase in 17- relative to 8-week-old nude mice [30]. In this study, we performed the YTS-1 xenograft experiment in 8- to 13-week old nude mice. Our findings suggest BNCT treatment induces an immune response by the host against tumor cells, triggering a strong cytotoxic reaction. Interestingly, Anxa1 expression in the tumor cell cytoplasm in IF7-^10^B drug-mediated BNCT groups remarkably increased relative to that seen in non-irradiated groups (Fig. 5a–f). Anxa1 protein has diverse functions in immunity and can be localized to the nucleus, cytoplasm, or cell surface [31]. It also plays a role in cancer chemotherapy [32–37]. For example, cell surface Anxa1 stimulates formyl-peptide receptor 1 (FPR1), which is implicated in anti-tumor immune responses elicited by anthracyclines or oxaliplatin [37]. Cisplatin-resistant lung cancer A549 cells have a twofold higher expression of Anxa1 localized to both the cell surface and cytoplasm, and Anxa1 knockdown increases sensitivity to cisplatin treatment [38]. A recent Phase I trial (NCT03784625) of melanoma-targeted radionuclide therapy showed that [^131^I]ICF01012 induces immunogenic tumor cell death, marked by a significant increase in cell surface Anxa1 and calreticulin [39]. This finding suggests that in our analysis of YTS-1 xenografts, inflammatory and immune responses that increase after BNCT upregulate Anxa1 expression in tumor tissues. Moreover, we hypothesize that if the first BNCT treatment upregulates Anxa1 in tumor tissues due to an inflammatory response or induced immunogenicity, more effective ^10^B accumulation in tumor tissues might occur following the second injection of IF7-^10^B drugs. Thus, multiple ultralow doses of IF7-^10^B drug-mediated BNCT likely boost BNCT therapeutic potential. Future studies should further examine mechanisms underlying Anxa1 upregulation in tumor tissue after BNCT. Although we did not observe a significant difference in the number of CD31-positive blood vessels per tumor area between groups, IF7-^10^B drug-mediated BNCT may destroy Anxa1-positive tumor vasculature as a way of boosting BNCT therapeutic potential (Fig. 6).

## Conclusions

In summary, here we have assessed the therapeutic potential of Anxa1-binding IF7-^10^B drug-mediated BNCT. Combining extremely efficient tumor vasculature targeting activity by IF7 with local radiation therapy such as BNCT could be an excellent dual targeting strategy. Further preclinical studies and Phase I clinical trials are needed to evaluate the clinical efficacy of ^10^B drugs conjugated to Anxa1-binding peptides in patients.

## Supplementary Information

**Additional file 1.**

## Data Availability

All data needed to evaluate the conclusions in the paper are present in the paper and/or the Supplementary Materials. Additional data related to this paper may be requested from the authors.

## Abbreviations

^10^BPA: *p*-Boronophenylalanine
^10^BSH: borocaptate sodium
Anxa1: Annexin A1
BNCT: Boron neutron capture therapy
LAT1: L-type amino acid transporter 1
PET: Positron emission tomography
PVA: Poly(vinyl alcohol)
