# Correction to: Tumor vasculature-targeted 10B delivery by an Annexin A1-binding peptide boosts effects of boron neutron capture therapy

**DOI:** 10.1186/s12885-021-07815-7

**Published:** 2021-01-29

**Authors:** Tohru Yoneyama, Shingo Hatakeyama, Mihoko Sutoh Yoneyama, Taku Yoshiya, Tsuyoshi Uemura, Takehiro Ishizu, Minoru Suzuki, Shingo Hachinohe, Shintaro Ishiyama, Motohiro Nonaka, Michiko N. Fukuda, Chikara Ohyama

**Affiliations:** 1 Department of Glycotechnology, Center for Advanced Medical Research, Hirosaki University Graduate School of Medicine, 5-Zaifu-cho, Hirosaki, 036-8562 Japan; 2 Department of Urology, Hirosaki University Graduate School of Medicine, 5-Zaifu-cho, Hirosaki, 036-8562 Japan; 3 Department of Cancer Immunology and Cell Biology, Oyokyo Kidney Research Institute, 90 Kozawa Yamazaki, Hirosaki, 036-8243 Japan; 4 Peptide Institute Inc., 7-2-9 Saito-Asagi, Osaka, Ibaraki 567-0085 Japan; 5 Particle Radiation Oncology Research Center, Institute for Integrated Radiation and Nuclear Science (KURNS), Kyoto University, 2-1010 Asashiro-nishi, Kumatori-cho, Sennan-gun, Osaka, 590-0494 Japan; 6 Aomori Prefecture Quantum Science Center (QSC), 2-190 Omotedate, Obuchi, Rokkasho-mura, Kamikita-gun, 039-3212 Japan; 7 Faculty of Science and Technology, Hirosaki University Graduate School of Science and Technology, 1-Bunkyo-cho, Hirosaki, 036-8562 Japan; 8 Department of Biological Chemistry, Human Health Sciences, Graduate School of Medicine, Kyoto University, 53 Shogoin-Kawahara-cho, Sakyo-ku, Kyoto, 606-8507 Japan; 9 Tumor Microenvironment and Cancer Immunology Program, NCI-Designated Cancer Center, Sanford Burnham Prebys Medical Discovery Institute, 10901 North Torrey Pines Road, La Jolla, CA 92037 USA

**Correction to: BMC Cancer (2021) 21:72**

**https://doi.org/10.1186/s12885-020-07760-x**

Following publication of the original article [1], the authors reported typesetting error in Figs. 3 and 4. Sections of the graphs were mistakenly omitted. The family name of Mihoko Sutoh Yoneyama was also incorrectly published. This has been amended in this correction article.

**Fig. 3:**
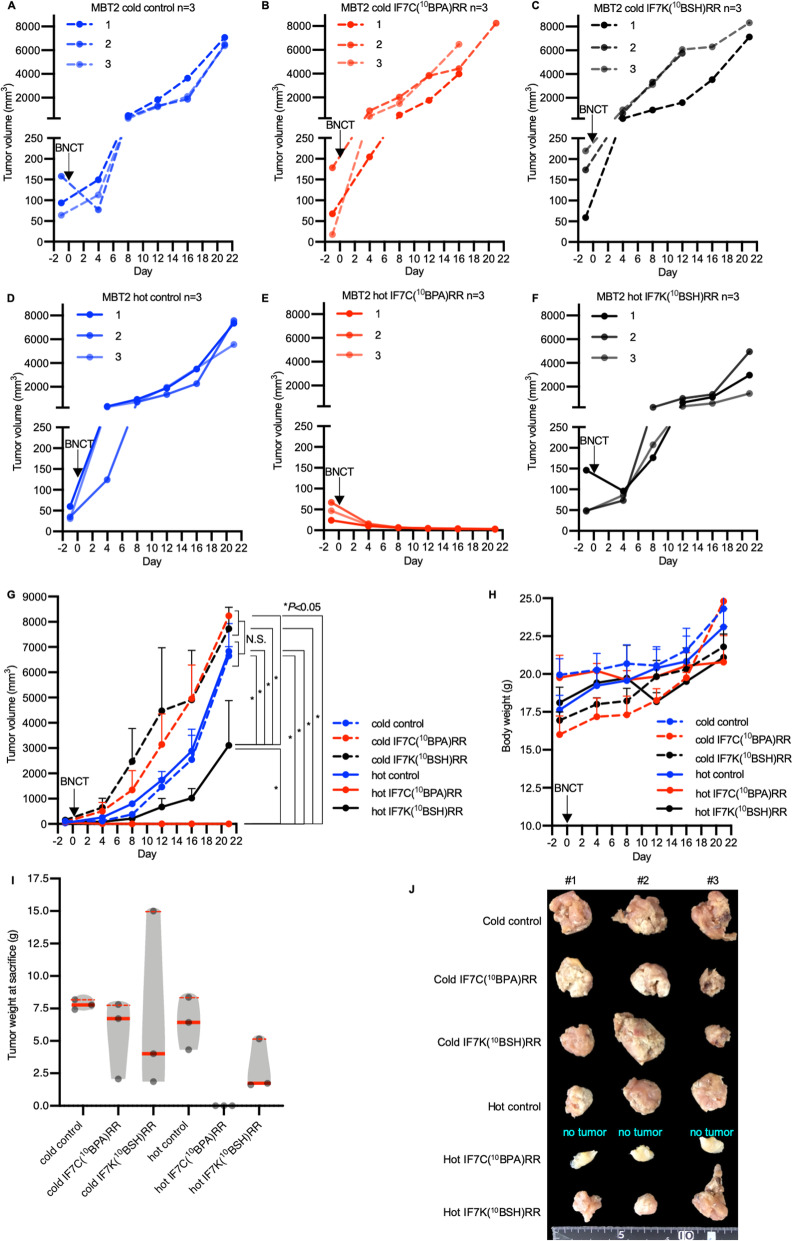
Antitumor effect of BNCT in murine MBT2 tumor-bearing mice. **a-f** Tumor growth curves from the following treatment groups: (**a**) untreated control (cold control, blue dashed line); (**b**) IF7C(^10^BPA)RR injection (cold IF7C(^10^BPA)RR, red dashed line); (**c**) IF7K(^10^BSH)RR injection (cold IF7K(^10^BSH)RR, black dashed line); (**d**) Neutron-irradiation (hot control, blue solid line); (**e**) IF7C(^10^BPA)RR-mediated BNCT (hot IF7C(^10^BPA)RR, red solid line); and **(f)** IF7K(^10^BSH)RR-mediated BNCT (hot IF7K(^10^BSH)RR, black solid line). Groups were intravenously injected, and tumors irradiated with epi/thermal neutrons 40 min after injection on day 1. **g** Tumor growth curve comparing groups analyzed in A-F. Results are expressed as means ± SD. *P < 0.05 (Holm–Sidak method). N.S.: no significant difference. **h** Body weight of indicated groups. Results are expressed as means ± SD. **i** Tumor weight of indicated groups at sacrifice. Results are expressed as violin plots with dot plots. Red bold lines indicate the median value, while red dashed lines indicate the interquartile range value (Mann–Whitney test) (**j**) Photograph of resected tumors from the injected right thighs. If a tumor completely shrank, whole right thighs were resected and labeled as “no tumor”

**Fig. 4:**
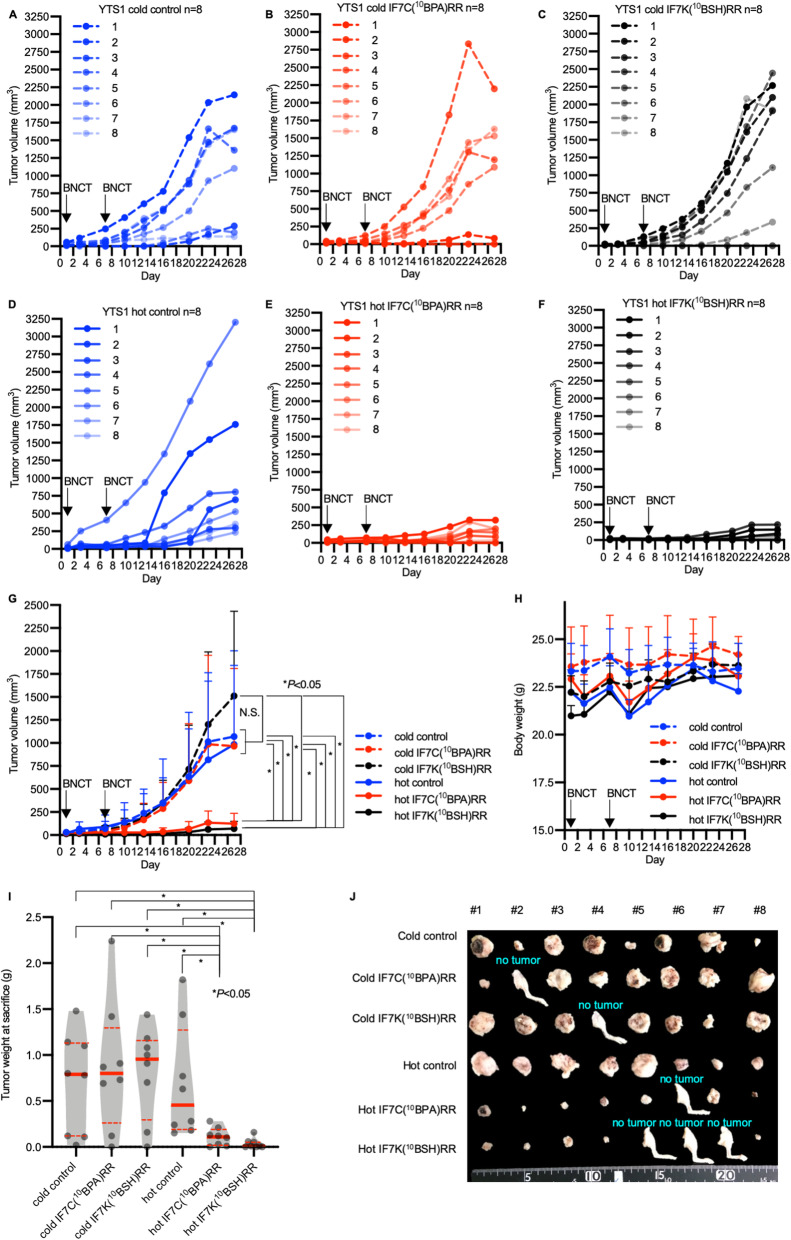
Antitumor effect of BNCT in a human YTS-1 xenograft model. **a-f** Tumor growth curves from the following treatment groups: (**a**) untreated control (cold control, blue dashed line); (**b**) IF7C(^10^BPA)RR injection (cold IF7C(^10^BPA)RR, red dashed line); (**c**) IF7K(^10^BSH)RR injection (cold IF7K(^10^BSH)RR, black dashed line); (**d**) Neutron-irradiation (hot control, blue solid line); (**e**) IF7C(^10^BPA)RR-mediated BNCT (hot IF7C(^10^BPA)RR, red solid line); and (**f**) IF7K(^10^BSH)RR-mediated BNCT (hot IF7K (^10^BSH)RR, black solid line). Indicated samples were intravenously injected, and tumors irradiated with epi/thermal neutrons 40 min after injection on days 1 and 7. **g** Tumor growth curve summarizing groups shown in A-F. Results are expressed as means ± SD. *P < 0.05 (Holm–Sidak method). N.S.: no significant difference. **h** Body weight of indicated groups. Results are expressed as means ± SD. **i** Tumor weight of indicated groups at sacrifice. Results are expressed as violin plots with dot plots. Red bold lines indicate the median value, while red dashed lines indicate the interquartile range. *P < 0.05 (Mann–Whitney test). (**j**) (**j**) Photograph of resected tumors from injected right thighs. If a tumor completely shrank, whole right thighs were resected and labeled as “no tumor”

The corrected Figs. 3 and 4 are given below. The original article [1] has been corrected.
