# Supplementary material for: Tumor vasculature-targeted 10B delivery by an Annexin A1-binding peptide boosts effects of boron neutron capture therapy
Source: BMC Cancer. 2021 Jan 15;21:72. doi: 10.1186/s12885-020-07760-x (PMC7809749; doi:10.1186/s12885-020-07760-x)
Supplement: Supplementary file 1 — Additional file 1. [file 12885_2020_7760_MOESM1_ESM.docx]

Supplementary Information for

Tumor vasculature-targeted ^10^B delivery by an Annexin A1-binding peptide boosts effects of boron neutron capture therapy.

Tohru Yoneyama^*, †, ‡‡^, Shingo Hatakeyama^†, ‡‡^, Mihoko Sutoh Yoneyama^‡^, Taku Yoshiya^§^, Tsuyoshi Uemura^§^, Takehiro Ishizu^§^, Minoru Suzuki^¶^, Shingo Hachinohe^#^, Shintaro Ishiyama^||^, Motohiro Nonaka^**^, Michiko N. Fukuda^††^, and Chikara Ohyama^†, §§^

^§§^ Chikara Ohyama

**Email:**  [coyama@hirosaki-u.ac.jp](mailto:coyama@hirosaki-u.ac.jp)

**This PDF file includes:**

Figures S1

Tables S1 to S2


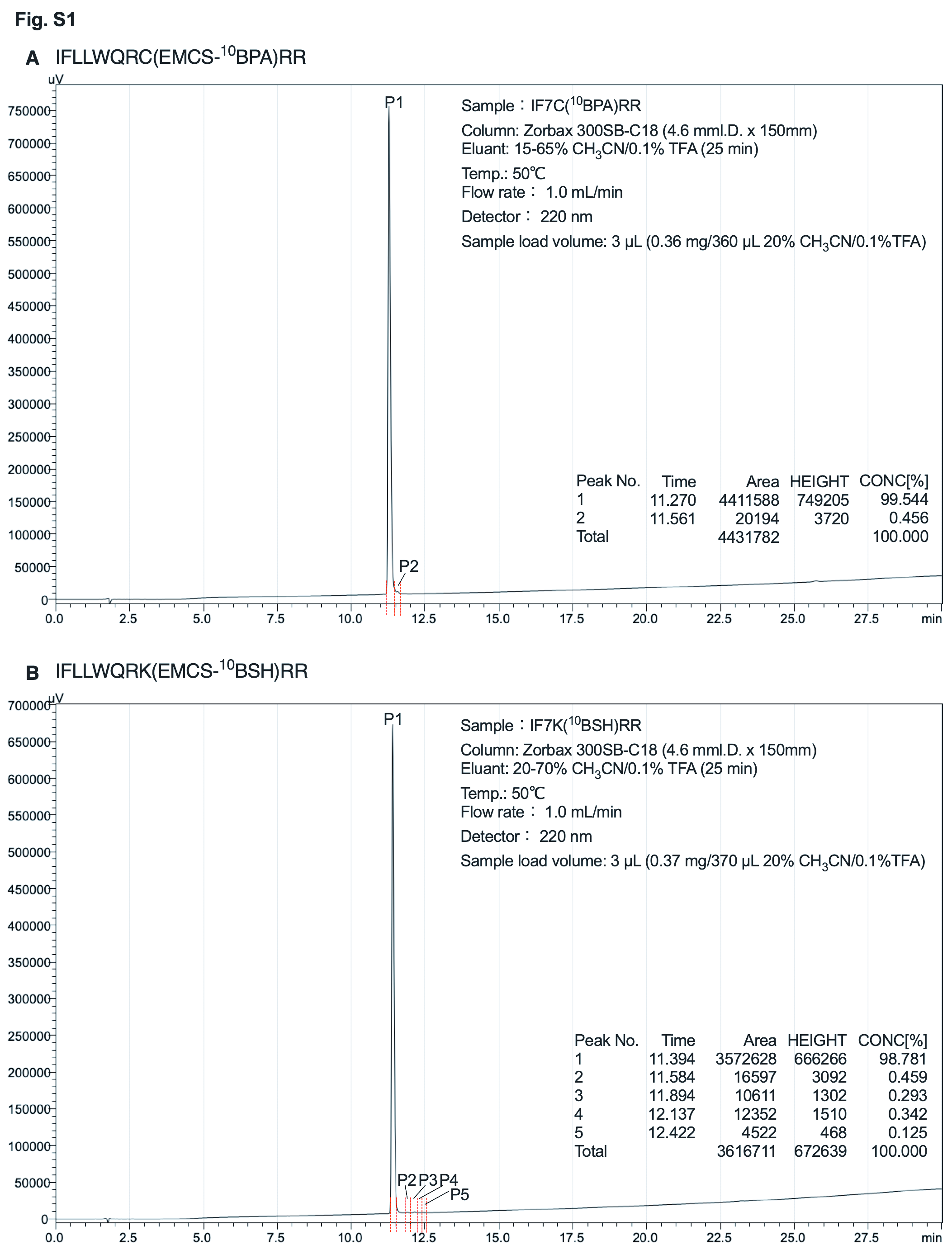


Fig. S1. HPLC chromatograph of purified IF7C(^10^BPA)RR and IF7K(^10^BSH)RR

**Table S1. Two-way ANOVA analysis of MBT2 tumor volume (Holm–Sidak method)**

| Holm–Sidak multiple comparisons test | | | | | |
| --- | --- | --- | --- | --- | --- |
| Day 1 |  |  | Mean diff. | Significant? | Adjusted *P* value |
| Cold IF7C(^10^BPA)RR | vs. | cold control | −17.27 | N.S. | > 0.9999 |
| Cold IF7K(^10^BSH)RR | vs. | cold control | 45.60 | N.S. | > 0.9999 |
| Hot control | vs. | cold control | −63.47 | N.S. | > 0.9999 |
| Hot IF7C(^10^BPA)RR | vs. | cold control | −59.52 | N.S. | > 0.9999 |
| Hot IF7K(^10^BSH)RR | vs. | cold control | −24.50 | N.S. | > 0.9999 |
| Cold IF7K(^10^BSH)RR | vs. | cold IF7C(^10^BPA)RR | 62.87 | N.S. | > 0.9999 |
| Hot control | vs. | cold IF7C(^10^BPA)RR | −46.19 | N.S. | > 0.9999 |
| Hot IF7C(^10^BPA)RR | vs. | cold IF7C(^10^BPA)RR | −42.25 | N.S. | > 0.9999 |
| Hot IF7K(^10^BSH)RR | vs. | cold IF7C(^10^BPA)RR | −7.227 | N.S. | > 0.9999 |
| Hot control | vs. | cold IF7K(^10^BSH)RR | −109.1 | N.S. | > 0.9999 |
| Hot IF7C(^10^BPA)RR | vs. | cold IF7K(^10^BSH)RR | −105.1 | N.S. | > 0.9999 |
| Hot IF7K(^10^BSH)RR | vs. | cold IF7K(^10^BSH)RR | −70.10 | N.S. | > 0.9999 |
| Hot IF7C(^10^BPA)RR | vs. | hot control | 3.947 | N.S. | > 0.9999 |
| Hot IF7K(^10^BSH)RR | vs. | hot control | 38.97 | N.S. | > 0.9999 |
| Hot IF7K(^10^BSH)RR | vs. | hot IF7C(^10^BPA)RR | 35.02 | N.S. | > 0.9999 |
|  |  |  |  |  |  |
| Day 4 |  |  | Mean diff. | Significant? | Adjusted *P* value |
| Cold IF7C(^10^BPA)RR | vs. | cold control | 389.9 | N.S. | 0.9997 |
| Cold IF7K(^10^BSH)RR | vs. | cold control | 533.7 | N.S. | 0.9989 |
| Hot control | vs. | cold control | 144.5 | N.S. | > 0.9999 |
| Hot IF7C(^10^BPA)RR | vs. | cold control | −100.4 | N.S. | > 0.9999 |
| Hot IF7K(^10^BSH)RR | vs. | cold control | −28.09 | N.S. | > 0.9999 |
| Cold IF7K(^10^BSH)RR | vs. | cold IF7C(^10^BPA)RR | 143.8 | N.S. | > 0.9999 |
| Hot control | vs. | cold IF7C(^10^BPA)RR | −245.4 | N.S. | > 0.9999 |
| Hot IF7C(^10^BPA)RR | vs. | cold IF7C(^10^BPA)RR | −490.3 | N.S. | 0.9992 |
| Hot IF7K(^10^BSH)RR | vs. | cold IF7C(^10^BPA)RR | −418.0 | N.S. | 0.9997 |
| Hot control | vs. | cold IF7K(^10^BSH)RR | −389.2 | N.S. | 0.9997 |
| Hot IF7C(^10^BPA)RR | vs. | cold IF7K(^10^BSH)RR | −634.1 | N.S. | 0.9973 |
| Hot IF7K(^10^BSH)RR | vs. | cold IF7K(^10^BSH)RR | −561.8 | N.S. | 0.9989 |
| Hot IF7C(^10^BPA)RR | vs. | hot control | −244.9 | N.S. | > 0.9999 |
| Hot IF7K(^10^BSH)RR | vs. | hot control | −172.6 | N.S. | > 0.9999 |
| Hot IF7K(^10^BSH)RR | vs. | hot IF7C(^10^BPA)RR | 72.29 | N.S. | > 0.9999 |
|  |  |  |  |  |  |
| Day 8 |  |  | Mean diff. | Significant? | Adjusted *P* value |
| Cold IF7C(^10^BPA)RR | vs. | cold control | 966.7 | N.S. | 0.6894 |
| Cold IF7K(^10^BSH)RR | vs. | cold control | 2091 | * | 0.0222 |
| Hot control | vs. | cold control | 421.3 | N.S. | 0.9437 |
| Hot IF7C(^10^BPA)RR | vs. | cold control | −373.6 | N.S. | 0.9437 |
| Hot IF7K(^10^BSH)RR | vs. | cold control | −164.0 | N.S. | 0.9437 |
| Cold IF7K(^10^BSH)RR | vs. | cold IF7C(^10^BPA)RR | 1125 | N.S. | 0.5754 |
| Hot control | vs. | cold IF7C(^10^BPA)RR | −545.4 | N.S. | 0.9339 |
| Hot IF7C(^10^BPA)RR | vs. | cold IF7C(^10^BPA)RR | −1340 | N.S. | 0.3632 |
| Hot IF7K(^10^BSH)RR | vs. | cold IF7C(^10^BPA)RR | −1131 | N.S. | 0.5754 |
| Hot control | vs. | cold IF7K(^10^BSH)RR | −1670 | N.S. | 0.1268 |
| Hot IF7C(^10^BPA)RR | vs. | cold IF7K(^10^BSH)RR | −2465 | ** | 0.0040 |
| Hot IF7K(^10^BSH)RR | vs. | cold IF7K(^10^BSH)RR | −2255 | * | 0.0108 |
| Hot IF7C(^10^BPA)RR | vs. | hot control | −794.9 | N.S. | 0.8227 |
| Hot IF7K(^10^BSH)RR | vs. | hot control | −585.3 | N.S. | 0.9339 |
| Hot IF7K(^10^BSH)RR | vs. | hot IF7C(^10^BPA)RR | 209.6 | N.S. | 0.9437 |
|  |  |  |  |  |  |
| Day 12 |  |  | Mean diff. | Significant? | Adjusted *P* value |
| Cold IF7C(^10^BPA)RR | vs. | cold control | 1682 | N.S. | 0.0824 |
| Cold IF7K(^10^BSH)RR | vs. | cold control | 3012 | *** | 0.0002 |
| Hot control | vs. | cold control | 273.7 | N.S. | 0.6706 |
| Hot IF7C(^10^BPA)RR | vs. | cold control | −1460 | N.S. | 0.1675 |
| Hot IF7K(^10^BSH)RR | vs. | cold control | −793.7 | N.S. | 0.5247 |
| Cold IF7K(^10^BSH)RR | vs. | cold IF7C(^10^BPA)RR | 1330 | N.S. | 0.1920 |
| Hot control | vs. | cold IF7C(^10^BPA)RR | −1408 | N.S. | 0.1740 |
| Hot IF7C(^10^BPA)RR | vs. | cold IF7C(^10^BPA)RR | −3142 | **** | < 0.0001 |
| Hot IF7K(^10^BSH)RR | vs. | cold IF7C(^10^BPA)RR | −2476 | ** | 0.0025 |
| Hot control | vs. | cold IF7K(^10^BSH)RR | −2738 | *** | 0.0007 |
| Hot IF7C(^10^BPA)RR | vs. | cold IF7K(^10^BSH)RR | −4472 | **** | < 0.0001 |
| Hot IF7K(^10^BSH)RR | vs. | cold IF7K(^10^BSH)RR | −3805 | **** | < 0.0001 |
| Hot IF7C(^10^BPA)RR | vs. | hot control | −1734 | N.S. | 0.0750 |
| Hot IF7K(^10^BSH)RR | vs. | hot control | −1067 | N.S. | 0.3448 |
| Hot IF7K(^10^BSH)RR | vs. | hot IF7C(^10^BPA)RR | 666.1 | N.S. | 0.5247 |
|  |  |  |  |  |  |
| Day 16 |  |  | Mean diff. | Significant? | Adjusted *P* value |
| Cold IF7C(^10^BPA)RR | vs. | cold control | 2413 | ** | 0.0031 |
| Cold IF7K(^10^BSH)RR | vs. | cold control | 2563 | ** | 0.0037 |
| Hot control | vs. | cold control | 261.4 | N.S. | 0.9147 |
| Hot IF7C(^10^BPA)RR | vs. | cold control | −2540 | ** | 0.0018 |
| Hot IF7K(^10^BSH)RR | vs. | cold control | −1522 | N.S. | 0.0789 |
| Cold IF7K(^10^BSH)RR | vs. | cold IF7C(^10^BPA)RR | 149.9 | N.S. | 0.9147 |
| Hot control | vs. | cold IF7C(^10^BPA)RR | −2152 | * | 0.0198 |
| Hot IF7C(^10^BPA)RR | vs. | cold IF7C(^10^BPA)RR | −4953 | **** | < 0.0001 |
| Hot IF7K(^10^BSH)RR | vs. | cold IF7C(^10^BPA)RR | −3936 | **** | < 0.0001 |
| Hot control | vs. | cold IF7K(^10^BSH)RR | −2302 | * | 0.0198 |
| Hot IF7C(^10^BPA)RR | vs. | cold IF7K(^10^BSH)RR | −5103 | **** | < 0.0001 |
| Hot IF7K(^10^BSH)RR | vs. | cold IF7K(^10^BSH)RR | −4085 | **** | < 0.0001 |
| Hot IF7C(^10^BPA)RR | vs. | hot control | −2801 | ** | 0.0016 |
| Hot IF7K(^10^BSH)RR | vs. | hot control | −1784 | N.S. | 0.0610 |
| Hot IF7K(^10^BSH)RR | vs. | hot IF7C(^10^BPA)RR | 1018 | N.S. | 0.3110 |
|  |  |  |  |  |  |
| Day 21 |  |  | mean diff. | Significant? | Adjusted *P* value |
| Cold IF7C(^10^BPA)RR | vs. | cold control | 1399 | N.S. | 0.4075 |
| Cold IF7K(^10^BSH)RR | vs. | cold control | 1279 | N.S. | 0.3555 |
| Hot control | vs. | cold control | 184.3 | N.S. | 0.9491 |
| Hot IF7C(^10^BPA)RR | vs. | cold control | −6642 | **** | < 0.0001 |
| Hot IF7K(^10^BSH)RR | vs. | cold control | −3534 | **** | < 0.0001 |
| Cold IF7K(^10^BSH)RR | vs. | cold IF7C(^10^BPA)RR | −119.8 | N.S. | 0.9491 |
| Hot control | vs. | cold IF7C(^10^BPA)RR | −1215 | N.S. | 0.4075 |
| Hot IF7C(^10^BPA)RR | vs. | cold IF7C(^10^BPA)RR | −8041 | **** | < 0.0001 |
| Hot IF7K(^10^BSH)RR | vs. | cold IF7C(^10^BPA)RR | −4933 | **** | < 0.0001 |
| Hot control | vs. | cold IF7K(^10^BSH)RR | −1095 | N.S. | 0.4075 |
| Hot IF7C(^10^BPA)RR | vs. | cold IF7K(^10^BSH)RR | −7921 | **** | < 0.0001 |
| Hot IF7K(^10^BSH)RR | vs. | cold IF7K(^10^BSH)RR | −4813 | **** | < 0.0001 |
| Hot IF7C(^10^BPA)RR | vs. | hot control | −6827 | **** | < 0.0001 |
| Hot IF7K(^10^BSH)RR | vs. | hot control | −3718 | **** | < 0.0001 |
| Hot IF7K(^10^BSH)RR | vs. | hot IF7C(^10^BPA)RR | 3109 | **** | < 0.0001 |

**Table S2.** **Two-way ANOVA analysis of YTS-1 tumor volume (Holm–Sidak method)**

| Holm–Sidak multiple comparisons test | | | | | |  |
| --- | --- | --- | --- | --- | --- | --- |
| Day 1 |  |  | Mean diff. | Significant? | Adjusted *P* value |  |
| Cold IF7C(^10^BPA)RR | vs. | cold control | −6.575 | N.S. | > 0.9999 |  |
| Cold IF7K(^10^BSH)RR | vs. | cold control | −10.01 | N.S. | > 0.9999 |  |
| Hot control | vs. | cold control | −11.73 | N.S. | > 0.9999 |  |
| Hot IF7C(^10^BPA)RR | vs. | cold control | −8.313 | N.S. | > 0.9999 |  |
| Hot IF7K(^10^BSH)RR | vs. | cold control | −17.30 | N.S. | > 0.9999 |  |
| Cold IF7K(^10^BSH)RR | vs. | cold IF7C(^10^BPA)RR | −3.438 | N.S. | > 0.9999 |  |
| Hot control | vs. | cold IF7C(^10^BPA)RR | −5.150 | N.S. | > 0.9999 |  |
| Hot IF7C(^10^BPA)RR | vs. | cold IF7C(^10^BPA)RR | −1.738 | N.S. | > 0.9999 |  |
| Hot IF7K(^10^BSH)RR | vs. | cold IF7C(^10^BPA)RR | −10.73 | N.S. | > 0.9999 |  |
| Hot control | vs. | cold IF7K(^10^BSH)RR | −1.713 | N.S. | > 0.9999 |  |
| Hot IF7C(^10^BPA)RR | vs. | cold IF7K(^10^BSH)RR | 1.700 | N.S. | > 0.9999 |  |
| Hot IF7K(^10^BSH)RR | vs. | cold IF7K(^10^BSH)RR | −7.288 | N.S. | > 0.9999 |  |
| Hot IF7C(^10^BPA)RR | vs. | hot control | 3.412 | N.S. | > 0.9999 |  |
| Hot IF7K(^10^BSH)RR | vs. | hot control | −5.575 | N.S. | > 0.9999 |  |
| Hot IF7K(^10^BSH)RR | vs. | hot IF7C(^10^BPA)RR | −8.987 | N.S. | > 0.9999 |  |
|  |  |  |  |  |  |  |
| Day 3 |  |  | Mean diff. | Significant? | Adjusted *P* value |  |
| Cold IF7C(^10^BPA)RR | vs. | cold control | −9.650 | N.S. | > 0.9999 |  |
| Cold IF7K(^10^BSH)RR | vs. | cold control | −16.35 | N.S. | > 0.9999 |  |
| Hot control | vs. | cold control | 25.66 | N.S. | > 0.9999 |  |
| Hot IF7C(^10^BPA)RR | vs. | cold control | −17.98 | N.S. | > 0.9999 |  |
| Hot IF7K(^10^BSH)RR | vs. | cold control | −22.96 | N.S. | > 0.9999 |  |
| Cold IF7K(^10^BSH)RR | vs. | cold IF7C(^10^BPA)RR | −6.700 | N.S. | > 0.9999 |  |
| Hot control | vs. | cold IF7C(^10^BPA)RR | 35.31 | N.S. | > 0.9999 |  |
| Hot IF7C(^10^BPA)RR | vs. | cold IF7C(^10^BPA)RR | −8.325 | N.S. | > 0.9999 |  |
| Hot IF7K(^10^BSH)RR | vs. | cold IF7C(^10^BPA)RR | −13.31 | N.S. | > 0.9999 |  |
| Hot control | vs. | cold IF7K(^10^BSH)RR | 42.01 | N.S. | > 0.9999 |  |
| Hot IF7C(^10^BPA)RR | vs. | cold IF7K(^10^BSH)RR | −1.625 | N.S. | > 0.9999 |  |
| Hot IF7K(^10^BSH)RR | vs. | cold IF7K(^10^BSH)RR | −6.613 | N.S. | > 0.9999 |  |
| Hot IF7C(^10^BPA)RR | vs. | hot control | −43.64 | N.S. | > 0.9999 |  |
| Hot IF7K(^10^BSH)RR | vs. | hot control | −48.63 | N.S. | > 0.9999 |  |
| Hot IF7K(^10^BSH)RR | vs. | hot IF7C(^10^BPA)RR | −4.988 | N.S. | > 0.9999 |  |
|  |  |  |  |  |  |  |
| Day 7 |  |  | Mean diff. | Significant? | Adjusted *P* value |  |
| Cold IF7C(^10^BPA)RR | vs. | cold control | −30.11 | N.S. | > 0.9999 |  |
| Cold IF7K(^10^BSH)RR | vs. | cold control | −17.23 | N.S. | > 0.9999 |  |
| Hot control | vs. | cold control | 18.09 | N.S. | > 0.9999 |  |
| Hot IF7C(^10^BPA)RR | vs. | cold control | −39.96 | N.S. | > 0.9999 |  |
| Hot IF7K(^10^BSH)RR | vs. | cold control | −60.09 | N.S. | > 0.9999 |  |
| Cold IF7K(^10^BSH)RR | vs. | cold IF7C(^10^BPA)RR | 12.89 | N.S. | > 0.9999 |  |
| Hot control | vs. | cold IF7C(^10^BPA)RR | 48.20 | N.S. | > 0.9999 |  |
| Hot IF7C(^10^BPA)RR | vs. | cold IF7C(^10^BPA)RR | −9.850 | N.S. | > 0.9999 |  |
| Hot IF7K(^10^BSH)RR | vs. | cold IF7C(^10^BPA)RR | −29.97 | N.S. | > 0.9999 |  |
| Hot control | vs. | cold IF7K(^10^BSH)RR | 35.31 | N.S. | > 0.9999 |  |
| Hot IF7C(^10^BPA)RR | vs. | cold IF7K(^10^BSH)RR | −22.74 | N.S. | > 0.9999 |  |
| Hot IF7K(^10^BSH)RR | vs. | cold IF7K(^10^BSH)RR | −42.86 | N.S. | > 0.9999 |  |
| Hot IF7C(^10^BPA)RR | vs. | hot control | −58.05 | N.S. | > 0.9999 |  |
| Hot IF7K(^10^BSH)RR | vs. | hot control | −78.17 | N.S. | > 0.9999 |  |
| Hot IF7K(^10^BSH)RR | vs. | hot IF7C(^10^BPA)RR | −20.12 | N.S. | > 0.9999 |  |
|  |  |  |  |  |  |  |
| Day 10 |  |  | Mean diff. | Significant? | Adjusted *P* value |  |
| Cold IF7C(^10^BPA)RR | vs. | cold control | −61.13 | N.S. | > 0.9999 |  |
| Cold IF7K(^10^BSH)RR | vs. | cold control | −41.46 | N.S. | > 0.9999 |  |
| Hot control | vs. | cold control | 0.4250 | N.S. | > 0.9999 |  |
| Hot IF7C(^10^BPA)RR | vs. | cold control | −112.2 | N.S. | > 0.9999 |  |
| Hot IF7K(^10^BSH)RR | vs. | cold control | −130.0 | N.S. | > 0.9999 |  |
| Cold IF7K(^10^BSH)RR | vs. | cold IF7C(^10^BPA)RR | 19.66 | N.S. | > 0.9999 |  |
| Hot control | vs. | cold IF7C(^10^BPA)RR | 61.55 | N.S. | > 0.9999 |  |
| Hot IF7C(^10^BPA)RR | vs. | cold IF7C(^10^BPA)RR | −51.03 | N.S. | > 0.9999 |  |
| Hot IF7K(^10^BSH)RR | vs. | cold IF7C(^10^BPA)RR | −68.91 | N.S. | > 0.9999 |  |
| Hot control | vs. | cold IF7K(^10^BSH)RR | 41.89 | N.S. | > 0.9999 |  |
| Hot IF7C(^10^BPA)RR | vs. | cold IF7K(^10^BSH)RR | −70.69 | N.S. | > 0.9999 |  |
| Hot IF7K(^10^BSH)RR | vs. | cold IF7K(^10^BSH)RR | −88.58 | N.S. | > 0.9999 |  |
| Hot IF7C(^10^BPA)RR | vs. | hot control | −112.6 | N.S. | > 0.9999 |  |
| Hot IF7K(^10^BSH)RR | vs. | hot control | −130.5 | N.S. | > 0.9999 |  |
| Hot IF7K(^10^BSH)RR | vs. | hot IF7C(^10^BPA)RR | −17.89 | N.S. | > 0.9999 |  |
|  |  |  |  |  |  |  |
| Day 13 |  |  | Mean diff. | Significant? | Adjusted *P* value |  |
| Cold IF7C(^10^BPA)RR | vs. | cold control | −72.40 | N.S. | 0.9999 |  |
| Cold IF7K(^10^BSH)RR | vs. | cold control | −51.54 | N.S. | > 0.9999 |  |
| Hot control | vs. | cold control | −49.85 | N.S. | > 0.9999 |  |
| Hot IF7C(^10^BPA)RR | vs. | cold control | −209.7 | N.S. | 0.9923 |  |
| Hot IF7K(^10^BSH)RR | vs. | cold control | −228.2 | N.S. | 0.9874 |  |
| Cold IF7K(^10^BSH)RR | vs. | cold IF7C(^10^BPA)RR | 20.86 | N.S. | > 0.9999 |  |
| Hot control | vs. | cold IF7C(^10^BPA)RR | 22.55 | N.S. | > 0.9999 |  |
| Hot IF7C(^10^BPA)RR | vs. | cold IF7C(^10^BPA)RR | −137.3 | N.S. | 0.9976 |  |
| Hot IF7K(^10^BSH)RR | vs. | cold IF7C(^10^BPA)RR | −155.8 | N.S. | 0.9976 |  |
| Hot control | vs. | cold IF7K(^10^BSH)RR | 1.687 | N.S. | > 0.9999 |  |
| Hot IF7C(^10^BPA)RR | vs. | cold IF7K(^10^BSH)RR | −158.2 | N.S. | 0.9976 |  |
| Hot IF7K(^10^BSH)RR | vs. | cold IF7K(^10^BSH)RR | −176.7 | N.S. | 0.9976 |  |
| Hot IF7C(^10^BPA)RR | vs. | hot control | −159.9 | N.S. | 0.9976 |  |
| Hot IF7K(^10^BSH)RR | vs. | hot control | −178.3 | N.S. | 0.9976 |  |
| Hot IF7K(^10^BSH)RR | vs. | hot IF7C(^10^BPA)RR | −18.45 | N.S. | > 0.9999 |  |
|  |  |  |  |  |  |  |
| Day 16 |  |  | Mean diff. | Significant? | Adjusted *P* value |  |
| Cold IF7C(^10^BPA)RR | vs. | cold control | −58.34 | N.S. | > 0.9999 |  |
| Cold IF7K(^10^BSH)RR | vs. | cold control | 1.325 | N.S. | > 0.9999 |  |
| Hot control | vs. | cold control | 2.650 | N.S. | > 0.9999 |  |
| Hot IF7C(^10^BPA)RR | vs. | cold control | −310.1 | N.S. | 0.7770 |  |
| Hot IF7K(^10^BSH)RR | vs. | cold control | −331.0 | N.S. | 0.7766 |  |
| Cold IF7K(^10^BSH)RR | vs. | cold IF7C(^10^BPA)RR | 59.66 | N.S. | > 0.9999 |  |
| Hot control | vs. | cold IF7C(^10^BPA)RR | 60.99 | N.S. | > 0.9999 |  |
| Hot IF7C(^10^BPA)RR | vs. | cold IF7C(^10^BPA)RR | −251.8 | N.S. | 0.8443 |  |
| Hot IF7K(^10^BSH)RR | vs. | cold IF7C(^10^BPA)RR | −272.6 | N.S. | 0.8177 |  |
| Hot control | vs. | cold IF7K(^10^BSH)RR | 1.325 | N.S. | > 0.9999 |  |
| Hot IF7C(^10^BPA)RR | vs. | cold IF7K(^10^BSH)RR | −311.4 | N.S. | 0.7770 |  |
| Hot IF7K(^10^BSH)RR | vs. | cold IF7K(^10^BSH)RR | −332.3 | N.S. | 0.7766 |  |
| Hot IF7C(^10^BPA)RR | vs. | hot control | −312.8 | N.S. | 0.7770 |  |
| Hot IF7K(^10^BSH)RR | vs. | hot control | −333.6 | N.S. | 0.7766 |  |
| Hot IF7K(^10^BSH)RR | vs. | hot IF7C(^10^BPA)RR | −20.85 | N.S. | > 0.9999 |  |
|  |  |  |  |  |  |  |
| Day 20 |  |  | Mean diff. | Significant? | Adjusted *P* value |  |
| Cold IF7C(^10^BPA)RR | vs. | cold control | −42.38 | N.S. | 0.9992 |  |
| Cold IF7K(^10^BSH)RR | vs. | cold control | 82.11 | N.S. | 0.9967 |  |
| Hot control | vs. | cold control | −39.75 | N.S. | 0.9992 |  |
| Hot IF7C(^10^BPA)RR | vs. | cold control | −568.9 | N.S. | 0.0533 |  |
| Hot IF7K(^10^BSH)RR | vs. | cold control | −602.0 | * | 0.0346 |  |
| Cold IF7K(^10^BSH)RR | vs. | cold IF7C(^10^BPA)RR | 124.5 | N.S. | 0.9951 |  |
| Hot control | vs. | cold IF7C(^10^BPA)RR | 2.625 | N.S. | 0.9992 |  |
| Hot IF7C(^10^BPA)RR | vs. | cold IF7C(^10^BPA)RR | −526.6 | N.S. | 0.0721 |  |
| Hot IF7K(^10^BSH)RR | vs. | cold IF7C(^10^BPA)RR | −559.6 | N.S. | 0.0542 |  |
| Hot control | vs. | cold IF7K(^10^BSH)RR | −121.9 | N.S. | 0.9951 |  |
| Hot IF7C(^10^BPA)RR | vs. | cold IF7K(^10^BSH)RR | −651.0 | * | 0.0166 |  |
| Hot IF7K(^10^BSH)RR | vs. | cold IF7K(^10^BSH)RR | −684.1 | ** | 0.0099 |  |
| Hot IF7C(^10^BPA)RR | vs. | hot control | −529.2 | N.S. | 0.0721 |  |
| Hot IF7K(^10^BSH)RR | vs. | hot control | −562.3 | N.S. | 0.0542 |  |
| Hot IF7K(^10^BSH)RR | vs. | hot IF7C(^10^BPA)RR | −33.08 | N.S. | 0.9992 |  |
|  |  |  |  |  |  |  |
| Day 23 |  |  | Mean diff. | Significant? | Adjusted *P* value |  |
| Cold IF7C(^10^BPA)RR | vs. | cold control | −26.20 | N.S. | 0.9211 |  |
| Cold IF7K(^10^BSH)RR | vs. | cold control | 186.9 | N.S. | 0.8673 |  |
| Hot control | vs. | cold control | −194.6 | N.S. | 0.8673 |  |
| Hot IF7C(^10^BPA)RR | vs. | cold control | −878.9 | *** | 0.0001 |  |
| Hot IF7K(^10^BSH)RR | vs. | cold control | −950.7 | **** | < 0.0001 |  |
| Cold IF7K(^10^BSH)RR | vs. | cold IF7C(^10^BPA)RR | 213.1 | N.S. | 0.8673 |  |
| Hot control | vs. | cold IF7C(^10^BPA)RR | −168.4 | N.S. | 0.8673 |  |
| Hot IF7C(^10^BPA)RR | vs. | cold IF7C(^10^BPA)RR | −852.7 | *** | 0.0002 |  |
| Hot IF7K(^10^BSH)RR | vs. | cold IF7C(^10^BPA)RR | −924.5 | **** | < 0.0001 |  |
| Hot control | vs. | cold IF7K(^10^BSH)RR | −381.5 | N.S. | 0.3341 |  |
| Hot IF7C(^10^BPA)RR | vs. | cold IF7K(^10^BSH)RR | −1066 | **** | < 0.0001 |  |
| Hot IF7K(^10^BSH)RR | vs. | cold IF7K(^10^BSH)RR | −1138 | **** | < 0.0001 |  |
| Hot IF7C(^10^BPA)RR | vs. | hot control | −684.3 | ** | 0.0053 |  |
| Hot IF7K(^10^BSH)RR | vs. | hot control | −756.1 | ** | 0.0016 |  |
| Hot IF7K(^10^BSH)RR | vs. | hot IF7C(^10^BPA)RR | −71.78 | N.S. | 0.9211 |  |
|  |  |  |  |  |  |  |
| Day 27 |  |  | Mean diff. | Significant? | Adjusted P value |  |
| Cold IF7C(^10^BPA)RR | vs. | cold control | −103.6 | N.S. | 0.9737 |  |
| Cold IF7K(^10^BSH)RR | vs. | cold control | 442.2 | N.S. | 0.1165 |  |
| Hot control | vs. | cold control | −85.74 | N.S. | 0.9737 |  |
| Hot IF7C(^10^BPA)RR | vs. | cold control | −945.6 | **** | < 0.0001 |  |
| Hot IF7K(^10^BSH)RR | vs. | cold control | −1000 | **** | < 0.0001 |  |
| Cold IF7K(^10^BSH)RR | vs. | cold IF7C(^10^BPA)RR | 545.8 | * | 0.0384 |  |
| Hot control | vs. | cold IF7C(^10^BPA)RR | 17.84 | N.S. | 0.9737 |  |
| Hot IF7C(^10^BPA)RR | vs. | cold IF7C(^10^BPA)RR | −842.1 | *** | 0.0002 |  |
| Hot IF7K(^10^BSH)RR | vs. | cold IF7C(^10^BPA)RR | −896.5 | **** | < 0.0001 |  |
| Hot control | vs. | cold IF7K(^10^BSH)RR | −528.0 | * | 0.0431 |  |
| Hot IF7C(^10^BPA)RR | vs. | cold IF7K(^10^BSH)RR | −1388 | **** | < 0.0001 |  |
| Hot IF7K(^10^BSH)RR | vs. | cold IF7K(^10^BSH)RR | −1442 | **** | < 0.0001 |  |
| Hot IF7C(^10^BPA)RR | vs. | hot control | −859.9 | *** | 0.0001 |  |
| Hot IF7K(^10^BSH)RR | vs. | hot control | −914.3 | **** | < 0.0001 |  |
| Hot IF7K(^10^BSH)RR | vs. | hot IF7C(^10^BPA)RR | −54.40 | N.S. | 0.9737 |  |
